# Nano‐biotechnology in tumour and cancerous disease: A perspective review

**DOI:** 10.1111/jcmm.17677

**Published:** 2023-02-24

**Authors:** Ambikesh Soni, Manohar Prasad Bhandari, Gagan Kant Tripathi, Priyavand Bundela, Pradeep Kumar Khiriya, Purnima Swarup Khare, Manoj Kumar Kashyap, Abhijit Dey, Balachandar Vellingiri, Suresh Sundaramurthy, Arisutha Suresh, José M. Pérez de la Lastra

**Affiliations:** ^1^ School of Nanotechnology Rajiv Gandhi Proudyogiki Vishwavidyalaya Bhopal India; ^2^ Institute of Clinical and Preventive Medicine University of Latvia Riga Latvia; ^3^ Amity Stem Cell Institute, Amity Medical School Amity University Haryana Haryana India; ^4^ Department of Life Sciences Presidency University West Bengal Kolkata India; ^5^ Stem cell and Regenerative Medicine/Translational Research Department of Zoology School of Basic Sciences, Central University of Punjab Maulana Azad National Institute of Technology Bathinda India; ^6^ Department of Chemical Engineering Maulana Azad National Institute of Technology Madhya Pradesh Bhopal India; ^7^ Department of Energy Maulana Azad National Institute of Technology & M/s Eco Science & Technology Madhya Pradesh Bhopal India; ^8^ Biotecnología de macromoléculas Instituto de Productos Naturales y Agrobiología, (IPNA‐CSIC) San Cristóbal de la Laguna Spain

**Keywords:** dendrimers, drug delivery, gold nanoparticles, nano‐biotechnology, silver nanoparticles, tumour

## Abstract

In recent years, drug manufacturers and researchers have begun to consider the nanobiotechnology approach to improve the drug delivery system for tumour and cancer diseases. In this article, we review current strategies to improve tumour and cancer drug delivery, which mainly focuses on sustaining biocompatibility, biodistribution, and active targeting. The conventional therapy using cornerstone drugs such as fludarabine, cisplatin etoposide, and paclitaxel has its own challenges especially not being able to discriminate between tumour versus normal cells which eventually led to toxicity and side effects in the patients. In contrast to the conventional approach, nanoparticle‐based drug delivery provides target‐specific delivery and controlled release of the drug, which provides a better therapeutic window for treatment options by focusing on the eradication of diseased cells via active targeting and sparing normal cells via passive targeting. Additionally, treatment of tumours associated with the brain is hampered by the impermeability of the blood–brain barriers to the drugs, which eventually led to poor survival in the patients. Nanoparticle‐based therapy offers superior delivery of drugs to the target by breaching the blood–brain barriers. Herein, we provide an overview of the properties of nanoparticles that are crucial for nanotechnology applications. We address the potential future applications of nanobiotechnology targeting specific or desired areas. In particular, the use of nanomaterials, biostructures, and drug delivery methods for the targeted treatment of tumours and cancer are explored.

## 1 INTRODUCTION

Despite the terms development and research were not yet very common, the American physicist Feynman pointed out the possibility of working at the nanoscale, and even at the smaller scale when he pronounced his famous conference “There is plenty of room at the bottom” in 1959. The inclusion of chemical, biological, and physical properties in the study of small particles, ranging from nanometres such as proteins, DNA, viruses to micrometeres, led to the concept of nanobiotechnology. Nanotechnology is a highly interdisciplinary field that combines biology and physics to produce multifunctional systems and devices that are more sensitive, specialized, and functional.^1^, ^2^, ^3^ Surfaces and interfaces play a crucial role in the development of novel nanomaterials. As particles get smaller, the number of atoms on their surface grows in comparison to the number of atoms inside their volume. This means that nanoparticles can be more reactive, leading, for example, to the production of catalysts or fillers that are more effective in composites. Because of their increased surface energy, nanoparticles can interact and adhere to each other. If the building blocks of nanomaterials are synthesized so that parts of the surface are sticky whilst other parts are passive and nonsticky, then random Brownian motion in a liquid can cause the blocks to adhere to each other in predetermined patterns and form larger structures.^4^ This is the basic idea behind the so‐called “bottom‐up” methods of self‐organization.

**FIGURE 1:**
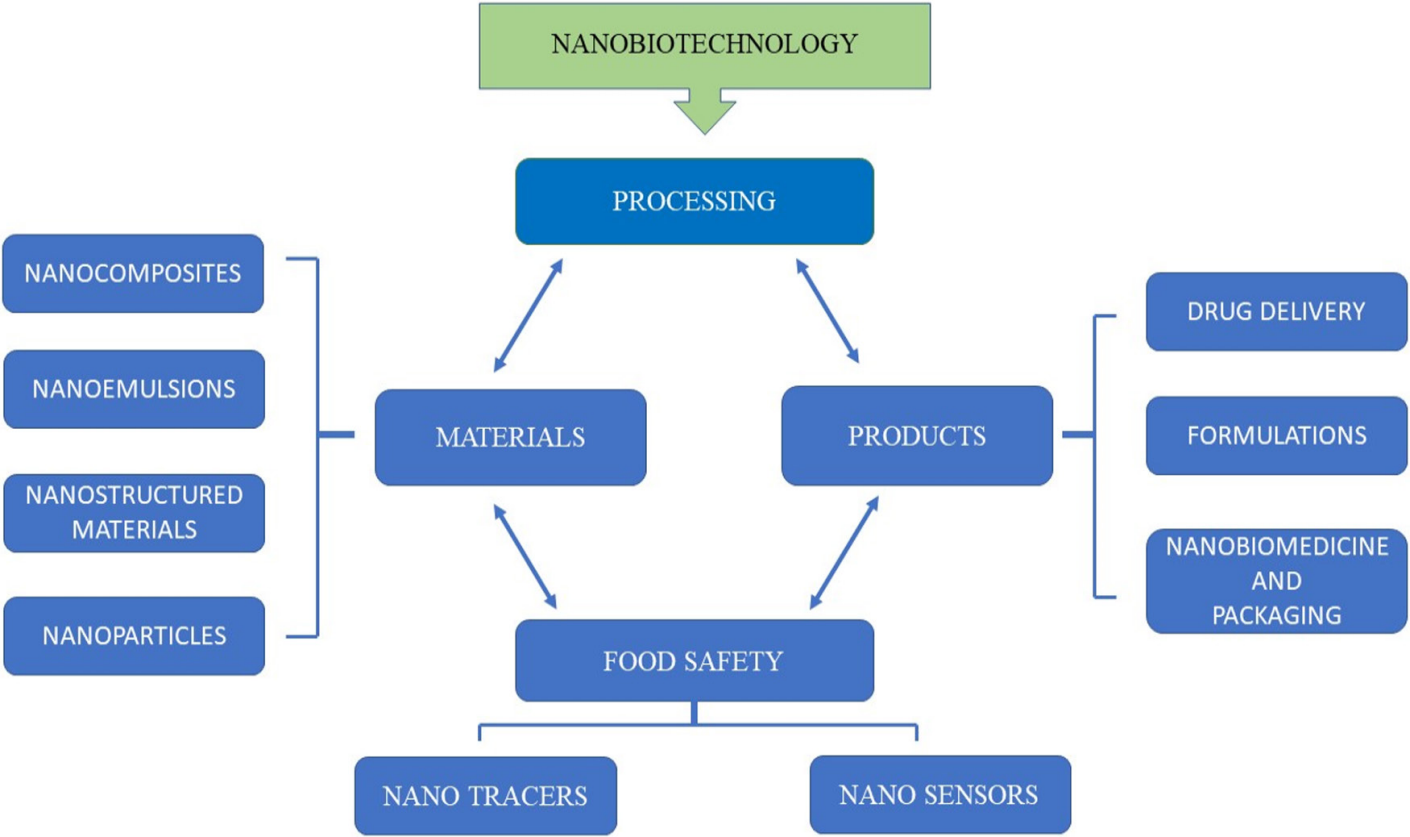
Representation of the significance and requirement of nano‐biotechnology in different field related to human life. As an interdisciplinary field of science, nanobiotechnology holds potential to develop improved drug delivery, formulation and packaging systems.

Amorphous structures differ from crystalline structures in the absence of long‐range atomic order and rotational and translational symmetry. Researchers have used nanocomposite materials to deliver drugs or genes to patients in the form of specific drugs to extend the half‐life of the drug. The biocompatibility of the nanomaterial can be improved by folding in the amorphous side of the nanomaterial, which is transported to the appropriate site to treat the disease.^3^ The liver, spleen and bone marrow are able to filter out nanoparticles larger than 200 nm efficiently; however, the particle size range of 10–200 nm is suitable for the circulation of spherical carriers.^5^, ^6^ Nanoparticles smaller than 10 nm can easily pass through the kidneys and enter the bloodstream by extravasation.^7^ The relationship between the size and shape of nanoparticles is closely related to their beneficial properties. Nanoparticles with large spherical shapes improve the flexibility of the required scaffold formed with drugs for treating specific diseases. A lot of credit goes to the advances in nanofabrication techniques, many nanoparticles with different physical, geometric and chemical properties have been produced in recent years.^8^ The significance of nanobiotechnology in human life has been shown in Figure 1.

**FIGURE 2:**
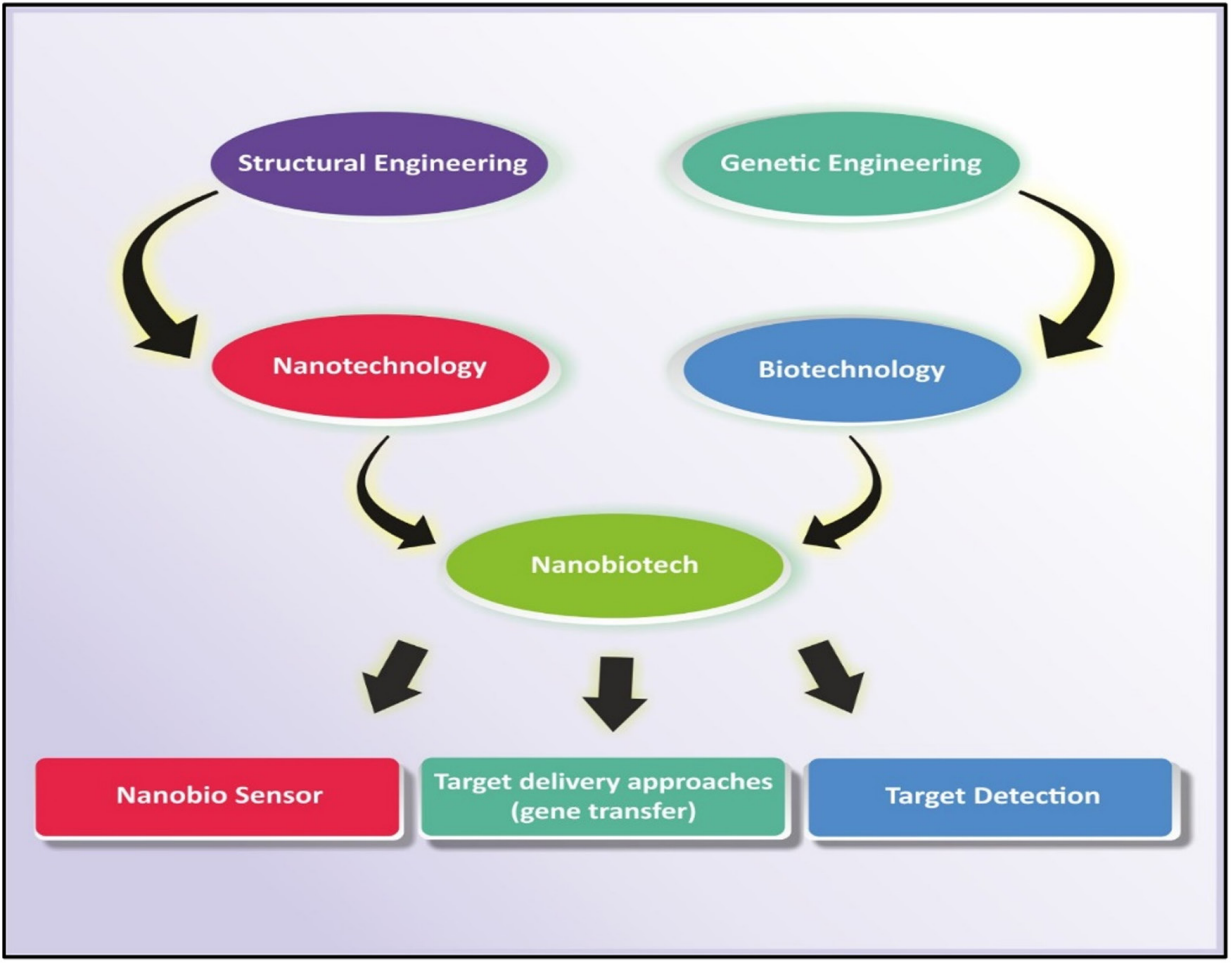
Diagram showing the involvement of nanotechnology along with biotechnology. The application of the amalgamation of nanotechnology and biotechnology is widespread as it involves other field's involvement such as structural and genetic engineering as well. The applications of nanotechnology are enormous in making nano biosensors, in target‐specific delivery of drugs and even in tumour/disease‐specific identification of targets as well.

Nanobiotechnology can act as a shield for various biomedical applications, as shown in Figure 2, which in turn is very useful for the living organism against diseases such as tumours or cancer.^9^ The use of various bio‐nanoparticles and nanomaterial's for drug delivery at the site of action under controlled application of desired drugs can be used for the treatment of numerous diseases or syndromes. The biocompatibility and bio‐adaptability of the nanomaterial's used are extremely important, and they pave the way for more precise medical research using nanotechnology and biotechnology.^10^, ^11^

In this review article, we provide a general overview of the aspects of nanoparticles that act as an essential component of nanotechnology. We discuss future aspects of nanobiotechnology to target desired or specific sites, in particular, the use of nanomaterials, biostructures, and drug delivery mechanisms for targeting tumours.

## 2 NANOPARTICLES—ESSENTIAL COMPONENT OF NANOTECHNOLOGY

Nanotechnology enables the development and use of structures and devices with organizational features ranging from single molecules to about 100 nm in size, a scale at which unique capabilities emerge compared to bulk materials. This refers to the ability to create yet another kind of nanostructures and devices capable of performing specific activities at the atomic and molecular levels. Nanotechnology is often considered the most promising emerging technology of the 21st century because it combines chemistry, biology, physics, materials science and engineering at the nanoscale. Other areas fighting for this distinction include biotechnology and information technology.^12^, ^13^, ^14^ In virtually all of these technologies, the strategy of combining different domains to formulate new materials involves the control of matter at the nanoscale. Nanoparticle fabrication is a critical component of nanotechnology science. Nanoparticles are specifically produced in the form of nanocrystals or nanosheets and are most often used to create nanostructured materials. The assembly of precursor particles and associated structures also plays a crucial role.^15^, ^16^

### 2.1 Why nanoparticles?

In the production of nanoparticles, crystals, and nanosheets, three types of properties are sought that offer different advantages:

- Size scaling, such as size confinement of certain flow structures, particle interfaces, and crystallizations, offers new physical, chemical, or biological properties. To distinguish smaller particles from larger interfaces, researchers have studied properties based on optical, electronic or quantum electromagnetic interactions.^17^, ^18^
- The magnetic and optoelectronic properties of the fabricated nanomaterials. The particle size and the incorporation of various artificial substances into the cells can be monitored, for example, on the basis of the colour changes in the suspensions.^19^, ^20^
- The molecular, atomic and microstructural structures. For example, various methods can be used to generate materials, including chemical self‐assembly, nanofabrication (3‐D structural manipulation), chemical generation of surface nanostructures, 3‐D molecular folding, bio‐templating, and various revolutionary approaches.^21^, ^22^

## 3 NANOMATERIALS

The physical and chemical properties of nanomaterials are determined by their particular composition, shape and size. The effects of nanomaterials on health and the environment also depend on their size, shape, etc. It is difficult to find a single, universally accepted definition of nanoparticles, and the scientific community is currently debating a strict definition of nanomaterials. According to the EU Commission's definition, a nanomaterial is “a man‐made or natural material containing unbound, aggregated or agglomerated particles with an external diameter between 1 and 100 nm”. There are four types of nanomaterials: (1) carbon‐based nanomaterials, (2) organic‐based nanomaterials, (3) composite‐based nanomaterials and (4) inorganic‐based nanomaterials. Inorganic‐based nanomaterials include various metal and metal oxide nanomaterials.^23^

The synthesis of inorganic nanomaterials such as metals and metal oxides using the “green synthesis technique” has gained popularity due to its sustainability, reliability, low cost, simple procedure, large‐scale production and harmlessness. In this method, inorganic nanomaterials can be produced by using a plant or plant extract as a reducing agent to reduce the metal precursor to its elemental form at the nanoscale.^24^, ^25^, ^26^, ^27^, ^28^, ^29^, ^30^

Inorganic nanoparticles include metals and metal oxides. Metal oxide‐based inorganic nanomaterials include zinc oxide (ZnO), copper oxide (CuO), magnesium aluminium oxide (MgAl_2_O_4_), titanium dioxide (TiO_2_), cerium oxide (CeO_2_), iron oxide (Fe_2_O_3_), silicon dioxide (SiO_2_), iron (Fe_3_O_4_), etc.^24^ Graphene, fullerene, single and multi‐walled carbon nanotubes, carbon fibres, activated carbon, and carbon black are all examples of carbon‐based nanomaterials.^31^ Examples of organic nanomaterials that do not contain carbon include dendrimers, cyclodextrin, liposomes, and micelles.^32^ Composite nanomaterials consist of any combination of metal‐based, metal oxide‐based, carbon‐based, and/or organic‐based nanomaterials. These nanomaterials have complicated structures, such as a metal–organic framework.

In the top‐down method, the materials are crushed by milling or grinding (Figure 1). This method is not suitable for the synthesis of symmetrically structured nanomaterials because the generation of microscopic particles requires a lot of energy. The fundamental disadvantage of this method is that the surface structure is not perfect, which directly affects the physical and surface properties of the materials produced. This strategy involves a variety of physical processes, including pyrolysis, atomization, electrospinning, laser ablation, sputtering, lithography, radiation‐induced chemical etching, and lithography.^33^, ^34^

Nanomaterials can be synthesized by bottom‐up and top‐down techniques, depending on the chemical and physical properties of the nanomaterials. Different size‐volume ratios can be achieved to perform desired functions with variable morphological properties.^35^, ^36^

In the bottom‐up technique, materials are synthesized atom by atom, cluster by cluster, or molecule by molecule. As a result of atom‐by‐atom deposition, nanoscale materials or nanomaterials are formed (see Figure 1). This strategy provides homogeneous morphology, size, shape, and distribution, making it more advantageous than the previous strategy. Bottom‐up approaches for nanomaterials fabrication include hydrothermal, solvothermal, chemical vapour deposition, chemical reduction, template‐assisted, sol–gel, organic ligand‐assisted, biological, and polyol techniques.^37^, ^38^ The various materials produced by nanotechnology are the results of colloidal science and interfacial science and contain nanorods, fullerenes, graphene, carbon nanotubes and various other nanoparticles. Fullerene is a family of nanomaterials with a size of 1/10 or even less in one of the three dimensions.^39^

### 3.1 Carbon‐based nanomaterials

Carbon has a variety of forms, including allotropes, which are structures with different dimensions. The bonds of carbon vary from sp3 of diamond, with a cubic face structure, to sp2 of graphite, with a hexagonal structure and fullerenes. In addition, the states of carbon vary depending on whether the carbon is amorphous or crystalline. This directly indicates whether or not the thermal, mechanical, and electrical performance of carbon lattice structures improves.^40^, ^41^ Diamond, for example, has a *face‐centred cubic structure* with a bond length of 1.54 Å and a lattice constant of 3.54 Å. Due to its extremely rigid lattice structure, diamond has the highest weight fraction and thermal conductivity of all bulk materials.^42^ The highly stable form of carbon is graphene, which is thermodynamically stable under standard conditions. Graphene consists of a layered, planar organization with a hexagonal structure arranged in a honeycomb lattice with a bond length of 1.42 Å.^43^

Fullerene is the smallest arrangement consisting of hexagonal and pentagonal rings known as Buckminsterfullerene. It was discovered in 1985 after the name of Buckminsterfullerene. These buckminsterfullerene molecules are used in a variety of studies and fields, such as drug delivery, solar cells, contrast agents for X‐ray imaging, superconductors, etc. One of the advantages is that it can be made in different shapes and sizes, varying from 30 to 3000 atoms of carbon.^44^, ^45^

### 3.2 Organic‐based nanomaterials

Nanoparticles possess all three dimensions but have properties that differ from those of bulk substances. The use of nanocarriers can be directly applied in biomedicine and as scaffolds for targeted drug delivery. Nanostructures have numerous applications and advantages, including surface reactivity, colloidal stability, and dispersion. The important functions of nanoparticles include active drug delivery and controlled release; nanoparticles are used together with tiny drugs, DNA and proteins to actively treat various diseases. According to the FDA and EMA (European Medicines Agency), biocompatible nanomaterials made of silk fibroin and chitosan are used in various biological applications.^46^, ^47^

Fibres with a diameter on the nanometre scale are called nanofibers. In the textile industry, this category is often expanded to include microfibers, which are fibres with a diameter of 1000 nm or less. In general, there are several ways to produce fibres, including melting, interfacial polymerization, electrospinning, antisolvent‐induced polymer precipitation, and electrostatic spinning. Carbon nanofibers, on the other hand, are fibres that have been graphitized by catalytic synthesis.^48^ The diameters of nanofibers depend on the type of polymer and the manufacturing technique. Compared to microfibers, all polymer nanofibers are characterized by their large surface‐to‐volume ratio, high porosity, high mechanical strength, and flexibility in functionalization. Nanofibers have found significant utility as scaffolds for biodegradable polymers in tissue engineering.^49^

### 3.3 Composite‐based nanomaterials

Various materials, such as composite nanoparticles, have emerged as a result of technological and scientific innovations. Optical sensors, specific catalysts, metal–semiconductor junctions, and packaging through film modification are some of the applications of these nanoparticles. For example, anatase titanium dioxide nanoparticles can be synthesized using a new sol–gel process and then analysed using a variety of methods.^50^ The main advantage of using egg white proteins as gelling agents is that they provide long‐term stability to the nanoparticles by preventing aggregation of the particles.^51^ It has been reported that the egg white solution is reliable, whilst the green gelling agent is inexpensive, and that this matrix can be useful in the sol–gel process for the synthesis of small‐size nanoparticles.^51^, ^52^

### 3.4 Inorganic‐based nanomaterials

An inorganic nanomaterial (NM) may be composed of a metal or a non‐metal element and may take the form of an oxide, hydroxide, chalcogenide or phosphate molecule. Applications of these materials in modern society include electronics, photonics, chemical sensors and biosensors, and biomedical devices. For example, quantum dots, polystyrene, magnetic, ceramic and metallic nanoparticles that have a central core of inorganic materials and exhibit fluorescent, magnetic, electrical, and optical properties.^53^, ^54^

The term “quantum dots” refers to man‐made, nanoscale semiconducting crystals capable of transporting electrons. UV light causes them to emit coloured light. Microwave‐assisted colloidal synthesis is one method that can be used to produce these compounds. Their applications include photovoltaic cells, biological applications, LED displays, photodetectors, and photocatalysts.^55^

## 4 METAL INORGANIC NANOPARTICLES

Nanoparticles (NPs) of metals such as silver, gold, and platinum are usually very small (less than 50 nm) and have a large exposed surface area. Because of their small size, they are able to penetrate the capillaries of tissues and cells. Because they have a large surface area and their chemistry can be altered (tunable surface chemistry), they can accommodate a variety of drugs.^56^ Metallic nanoparticles have been used for the controlled release of drugs in the treatment of cancer.^57^, ^58^ The surface properties of NPs also affect the duration of blood circulation. After administration, NPs can bind to serum proteins known as opsonins. These include immunoglobulins and complement proteins that help macrophages recognize NPs. Thus, opsonization is a critical factor in whether NPs remain in the bloodstream or are phagocytosed by macrophages. Therefore, altering their surface is an effective technique to increase or decrease their retention time in the blood.^59^ Despite the goal that these metals should remain inert, bioaccumulation and toxicity may occur.^60^ Metallic NPs can be synthesized and provided with a variety of chemical functional groups so that they can be coupled with antibodies, ligands, and desired drugs.^61^ This results in a wide range of potential biotechnological applications, including magnetic separation, pre‐concentration of target analytes, delivery of target drugs, and carriers for gene therapy.^62^ One of the major advantages of metal NPs is their ability to absorb light energy and convert it efficiently into heat. Therefore, some of them can be used in hyperthermic tumour therapy, in which thermal energy is generated by light stimulation to improve the specificity of this therapy.^58^

Understanding the physical and chemical properties of inorganic nanoparticles is a critical component for their use. Characterization of inorganic nanomaterials is performed using a variety of methods and instruments, including UV–Vis spectroscopy, Raman spectroscopy, Fourier transform infrared spectroscopy (FTIR), field emission scanning electron microscope (FESEM), scanning electron microscope (SEM), high‐resolution transmission microscope (HRTEM) and transmission electron microscope (TEM). Powder X‐ray diffraction (PXRD) is used to identify the crystalline phase and crystal structure. The fraction of elements present is determined by energy dispersive spectroscopy (EDS). The particle size can be determined by X‐ray powder diffraction (PXRD) and dynamic light scattering (DLS). X‐ray photoelectron spectroscopy, often referred to as XPS, can be used to determine the oxidation state of the elements present and their elemental composition. The surface area of nanoparticles can be determined using the Brunauer–Emmett–Teller method (BET).^63^ In addition, various imaging techniques such as MRI, computed tomography, positron emission tomography, ULTS, surface‐enhanced Raman spectroscopy, and optical imaging have been developed to detect a variety of diseases, including cancer.^64^, ^65^

In recent years, oncology has paid much attention to inorganic nanoparticles (INPs) because of their enormous potential for drug delivery, gene therapy, photodynamic treatment (PDT), photothermal therapy (PTT), bioimaging, motion control and so on.^66^, ^67^, ^68^ Recently, attempts have been made to modify naked INPs to overcome their natural limitations, such as rapid elimination by the immune system, low accumulation at tumour sites and significant toxicity to the organism.^69^ Many scientists have used cell membrane‐camouflaged INPs (CMC‐INPs) to hide nanoparticles in the same way as cells. CMC‐INPs are as adaptable as normal cells because they retain the sticky surface molecules, receptors and functional proteins of the original cell membrane.^70^ With the current development of loading component technology, the incorporation of functional components for multimodal treatment in INPs will be a future trend.

To achieve self‐assembly, multifunctional INPs that mimic natural cell membranes generally undergo three steps: (1) isolation of the vesicles from the cell membrane, (2) synthesis of the INP cores and (3) finally assembly of the vesicles and the INPs into a core‐shell nanostructure. The third step is crucial because it determines how well your CMC‐INPs are made. The addition of cholesterol and divalent ions (such as MgCl_2_, CaCl_2_, etc.) to hypotonic buffers is being studied as a means of maintaining cell membrane integrity and reducing the loss of functional proteins. Mechanical stress during extrusion encourages INPs to cross the phospholipid bilayers and helps the cell membrane to wrap around the INPs, resulting in vesicle‐nanoparticle fusion due to the fluidity of the cell membrane. Sonication is a good substitute for co‐extrusion. Ultrasound waves can cause cavitation bubbles that disrupt membrane structure and facilitate the recombination of cell membrane fragments around INPs.^70^

Camouflaging INPs with red blood cell membranes (RBCM) may prolong the short half‐life of INPs and increase the biocompatibility of INPs, making INPs more suitable for drug delivery, tumour imaging, and other applications. However, the low targeting capacity of RBCM INPs limits their application in precision cancer treatment. Most often, it is leaky blood vessels that cause RBCM‐INPs to deposit in a tumour, but tumours are different and not all have leaky blood vessels.^71^, ^72^ Cancer cell membranes (CCMs) retain cell adhesion molecules from the surface of the parent cells, such as selectins, cadherins, immunoglobulin superfamily, integrins, and lymphocyte homing receptors. CCM‐INPs are able to evade immune defence and remain undetected in the bloodstream because they express self‐markers and self‐recognition molecules. This “homing” property enables drug‐loaded CCM‐INPs to exert higher antitumor activity in vivo. Coating CCM may therefore increase their residence time in the body and enhance the ability of INPs to target homologues. By tailoring the source of CCM on the NPs, this bionic method was able to correctly identify and treat different types of cancer.^72^ However, the production of CMC‐INP is a lengthy and costly process that must be performed under sterile conditions to ensure the quality of the final product. At the same time, the limited shelf life of CMC‐INP makes it difficult to meet the requirements of clinical applications.

In addition to tumour antigens, adjuvants are also required for efficient immune activation. To elicit tumour‐specific immune responses, Fang et al developed a biomimetic nanovaccine coated with the membrane of melanoma cells (B16‐F10). The result was that the composite NPs could induce the immune system to better target cancer cells.^73^

Although the results of many CCM‐INPs systems are promising, there are still some obstacles to overcome before these technologies can be commercialized. People may have concerns about injecting cancer cell‐derived compounds into their bodies. This is especially true for people who are at risk for certain cancers and could use this technique as a preventive vaccine. As long as there is a hope of eliminating even a single malignancy through therapy or vaccination, many different research strategies will remain active towards CCM‐INPs.^70^ The INPs can overcome the limitations of platelets in tumour treatment by stably taking over the functions of platelets, such as immunological evasion and aggregation of inflammatory foci. The encapsulation of platelet‐derived membranes (PLTMs) has the advantage that they can be delivered directly to the site of inflammation or tumour growth, unlike the membranes of red blood cells. Researchers have taken advantage of this property to deliver drugs and INPs to the tumour site. NPs encapsulated in platelets use both passive and active targeting mechanisms to exert their therapeutic effects.^74^, ^75^ Platelet membrane‐camouflaged NPs (PNPs) use autologous antigens produced by PLTM, such as CD47, on their surface ligands to bypass clearance by the immune system and deliver more drugs to sites of inflammation.^76^ Several surface receptors on PNPs can actively interact with targeted components of cancer tissues. Overexpressed CD44 on the surface of tumour cells can be recognized by cell adhesion molecules such as P‐selectin glycoprotein ligand‐1 (PSGL‐1). All of these synergies give PNPs their potent tumour‐targeting capabilities.^72^

Reliance on passive targeting is greater than on active targeting. Pei et al. increased the precision of drug delivery using PLTM. They emulsified IR780 (a common NIR fluorescent dye), PLGA, and DOX to form IR780@PLGA/DOX NPs for the treatment of breast cancer. NPs with drugs and photothermal agents wrapped in natural PLTM were not recognized and degraded by the immune system.^77^ To take advantage of the long circulation of PLTM and its ability to target cancer, Liu et al. have fabricated PLTM‐camouflaged magnetic Fe_3_O_4_‐NPs (PLTM‐MNs) containing the tumour‐targeting molecules of PLTM and the photothermal transformation and magnetic responses of Fe_3_O_4_‐NPs. Studies using inductively coupled plasma atomic emission spectrometers on mouse tissues showed that PLTM MNs had lower uptake ability and better tumour targeting. The results suggest that PLTMs could be used to improve PTT in cancer treatment.^66^

PLTM‐INPs are not only used to encapsulate small molecule anti‐cancer INPs such as DOX and Fe_3_O_4_ NPs, but also to deliver genes to enable gene therapy in cancer. One example is the use of PLTM‐coated small interfering RNA (siRNA) to form P‐MOF siRNA, which is then used to knock down tumour‐related genes.^78^, ^79^ Inhibition of tumour growth and survival rates in mice treated with porous metal–organic skeleton (MOF)‐siRNA were much higher than in mice treated with bare INPs.^80^ PLTM‐INPs not only increase accumulation in tumour with CTC targeting potential, but also can reduce systemic toxicity.^81^

PLTM‐INPs have the potential to be one of the best options for biomimetic systems for cancer therapy because they are abundant, inexpensive, easy to extract, and minimally immunogenic. However, large‐scale production and successful clinical application require a standardized production process.^82^

### 4.1 Gold nanoparticles

Most nanoparticle‐based carriers are theoretically tagged with probes for tumour targeting. This is done with the goal of increasing the therapeutic efficacy of anticancer drugs whilst improving their targeting of the tumor.^83^ Gold nanoparticles (GNPs) range in size from 2 to 100 nm, with particles 20–50 nm in size having the highest cellular absorptive capacity. 40–50 nm‐sized particles have been shown to have specific cytotoxicity. GNPs are characterized by the fact that they promote drug efficacy, can be loaded with drugs, are biocompatible and can easily reach the target site with the bloodstream, are not cytotoxic to normal cells, and can be produced by various methods.^84^ Due to the advantages of functionalized GNPs, their use has expanded and they could be used in a variety of applications, from the manufacturing of photonic devices to the detection of organic and biomolecules to charge storage systems.^85^

There are numerous types of GNPs, including gold nanorods, gold nanocages and gold nanospheres.^86^ Previous research has suggested that the ideal size of nanocarriers for tumour targeting should be between 10 nm and 200 nm, with surface‐modified caution charges.^84^ These nanoscale carriers would be immediately removed from the reticuloendothelial system if there were no positively or negatively charged changes on the envelope surface.^86^ Therefore, the decoration of the surface of a nanocarrier with polyethylene glycol is the most commonly used surface coating agent to prevent and reduce clearance mediated by the reticuloendothelial system (PEG).^83^ GNPs can serve as contrast agents and dose enhancers in image‐guided nanoparticle‐enhanced radiotherapy using kilovoltage cone‐beam computed tomography for biomedical and cancer therapy applications.^84^

Since N‐oxyamide‐modified molecules are metabolically stable, coating GNPs with an N‐oxyamide‐linked glycoglycerolipid improves drug delivery to target tissues.^87^ Recently, researchers coated gold nanorods with a modified low molecular weight hyaluronic acid coupled with pH‐sensitive groups. The generated gold nanorods accumulated accumulate in tumour areas with acidic environments.^88^, ^89^ To increase the specificity of drug delivery, certain antibodies can be applied to the surface of GNPs. Cellular absorption of GNPs can be enhanced by coating them with hydrophilic polymers such as mercapto‐undecanesulfonate (MUS) or both MUS and octanethiol.^90^ Jia et al synthesized ‘bio‐friendly virus’‐like GNPs by scattering GNPs on the surface of polymeric substances, thereby changing the shape of the GNP complex to a virus‐like form and thereby increasing its uptake by human cervical cancer cells (HeLa), monkey renal fibroblast cells (COS7), human hepatoma cells (HepG2), and mouse fibroblasts (3T3).^91^ By incorporating molecules into the monolayer, such as antibodies (and their fragments), lectins, proteins, hormones or charged molecules, the nanoparticles can be targeted at the cell, tissue or organ level. Using an ultrasensitive electrochemical cytosensing platform, Zhang et al. produced GNP‐coated carbon nanospheres with an antibody for the carcinoembryonic antigen to diagnose non‐small cell lung cancer (NSCLC). In A549 lung cancer cells, the GNP complex showed no toxicity. This in vitro study provides evidence that the GNPs used may be able to detect NSCLC at an early stage.^92^, ^93^

Researchers have shown that they can identify and image cancer cells by conjugating anti‐EGFR to the surface of gold nanoparticles. The work by Patra et al showed that a multifunctional GNP labelled with C225 and gemcitabine, a drug commonly used in clinics to treat various cancers, can be effective.^94^ The proliferation of pancreatic cancer cells was slowed down with this treatment thanks to the GNPs.^95^ This approach has the potential to be used as a therapeutic cancer treatment against tumours in which EGFR is overexpressed.

Since cancer cells often have too much of the folic acid receptor on their surface, it is an important receptor for delivering drugs to the right site. Bhattacharya et al. used multifunctional GNPs as a potential treatment for ovarian cancer. Compared to normal cell lines, malignant cells (OV‐167, OVCAR 5) were more susceptible to the deleterious effects of gold nanoparticles in combination with thiolated polyethylene glycol (HS‐PEG), cisplatin,^96^ and folic acid (HUVECs, OSE).^97^, ^98^

For the clinical use of nanoparticles, a fundamental understanding of their behaviour in the tumour microenvironment is essential to improve their effectiveness as therapeutic and diagnostic agents. Cancer therapies usually have difficulty reaching the hypoxic core of the tumour because solid tumours are characterized by wide intercapillary distances and fluctuating blood flow. However, angiogenesis often occurs in the growing outer layer of a solid tumour. This highly vascularized area is notoriously leaky, creating a situation of increased permeability and retention. The hyperpermeability of solid tumours is exploited to develop nanoparticles for passive uptake.^99^ The angiogenic blood channels of this tissue may contain 600 nm gaps between endothelial cells through which carriers can extravasate. This results in a concentration of carrier at the tumour that is up to ten times higher than that of the same amount of free drug administered intravenously.^100^ It has been shown that intravenous injection of GNPs containing tumour necrosis factor (TNF) results in TNF being transported to a tumour in the colon in vivo.^101^ TNF bound to nanoparticles was less dangerous and more effective than native TNF.^102^

Surface plasmon resonance gives gold nanoparticles their optical properties. These properties can be used to treat diseased tissue with photodynamic therapy (PDT).^103^, ^104^ Using short laser pulses directed at monoclonal antibodies associated with light‐absorbing micro‐ and nanoparticles, researchers have developed a technique for selective cell targeting and photodynamic treatment.^105^ To prove the efficacy of gold nanoparticles in PTT, Lin et al used 30 nm gold nanospheres coupled with IgG antibodies. This in vitro study showed that 95% of CD8‐expressing lymphocytes were destroyed after treatment with GNPs and immunoglobulin (IgG).^106^

Using GNPs labelled with prostate cancer‐specific bombesin peptide analogues that generate beta radiation, it is possible to achieve a higher therapeutic dose. This design could potentially also be used for breast and small cell lung cancer cells, as the peptide analogue has a strong affinity for cell surface receptors.^107^

An important property of gold nanoparticles is that they do not oxidize or degrade in living organisms. These desirable properties are critical for the development of nanomaterials as drugs and diagnostics in the medical field. Radioactive GA ‐198 gold nanoparticles were administered to SCID mice in a single dose. After 3 weeks, researchers observed a remarkable 82% reduction in tumour size between the treatment and control groups. The high tolerability of GA ‐198 GNPs was also demonstrated by the fact that only 2% of the radioactivity reached non‐target organs and that blood levels returned to normal in the treatment group.^108^

When injected subcutaneously, intramuscularly, or topically, nanoparticles used as drug carriers usually have a longer residence time, typically in local lymph nodes, than the free drug. The biological distribution of nanoparticles strongly depends on the hydrodynamic radius and surface charge of the nanoparticles. Gold nanoparticles with a size of 10 nm were found in many organs, whilst those with a size of 15 nm or 50 nm were able to cross the blood–brain barrier. Citrate‐capped gold nanoparticles with a size of 15 nm had the highest penetration coefficient. The 100 nm and 200 nm particles remained on the surface of the skin, whilst the smaller particles were able to penetrate deeper into the dermis and subdermis of the skin.^109^

Understanding the basic mechanisms of interactions between nanoparticles and cells is critical for the application of nanotechnology in cancer therapies, screening, and diagnostics.

#### 4.1.1 Synthesis of GNPs

**FIGURE 3:**
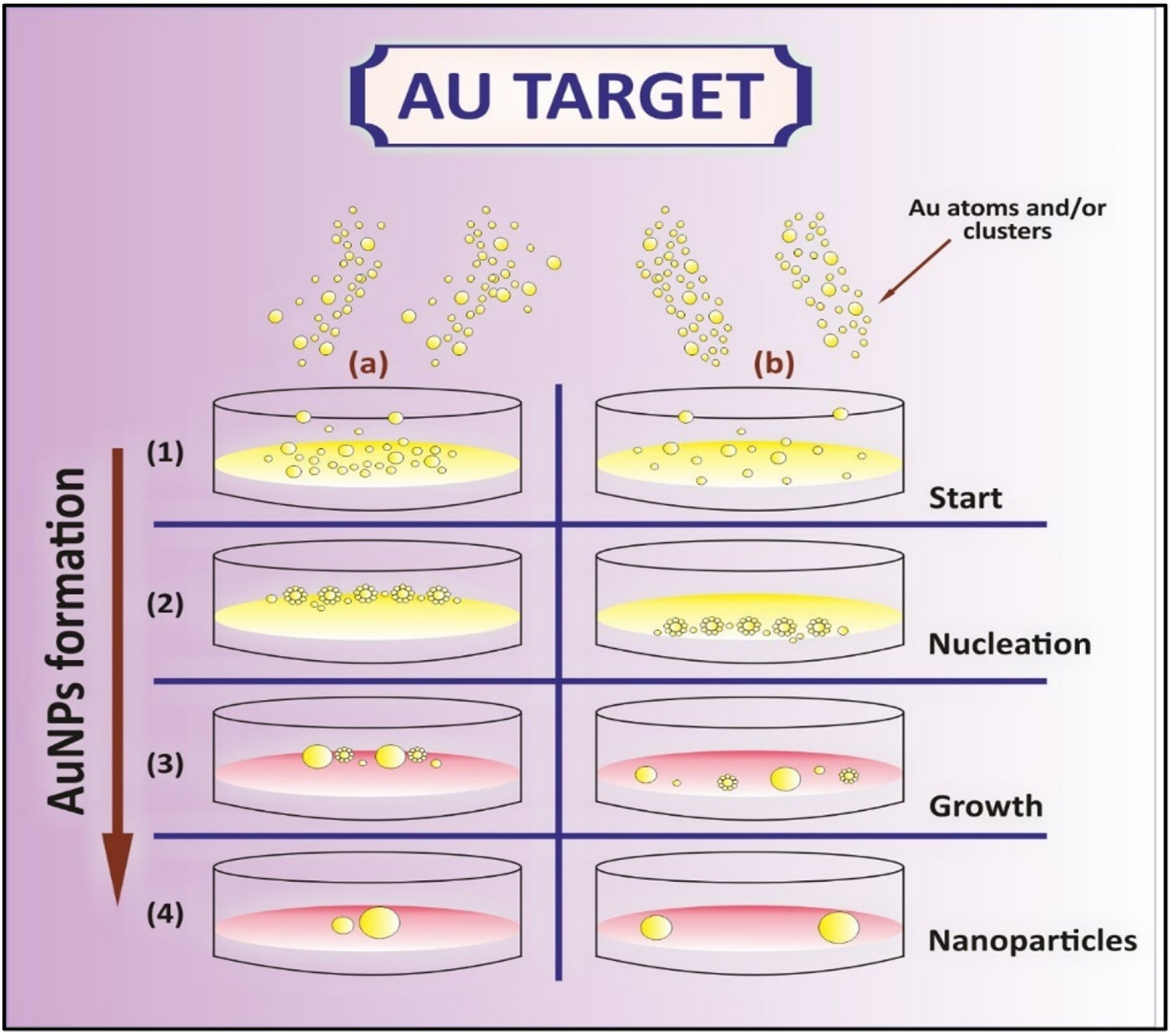
Steps showing the synthesis of gold nanoparticles (Au = Gold). (1) Bottom‐up approach or self‐assembly use forces like physical or chemical one at nanoscale to muster small units into larger one in case of the gold atoms and/or clusters, leading to the (2) nucleation phase to form a new structure by self‐assembly, leading to the (3) growth phase, where surface development occurs, leading to the (4) synthesis of GNPs.

GNPs can be produced using numerous technologies, including chemical and physical methods that encompass various physical and chemical methods, including the template approach, sonolysis, electrochemical method, green biosynthesis method, solvent‐free photochemical method, γ‐irradiation method, and hot injection technique.^110^, ^111^ In most cases, the preparation of gold nanoparticles involves the use of reducing agents in conjunction with the chemical reduction of chloroauric acid (HAuCl_4_).^112^ Figure 3 shows the stages for the synthesis of GNPs. (a) Beginning of the process as a bottom‐up method, leading to the (b) nucleation phase, i.e., the formation of a new phase or structure by self‐assembly methods, leading to the (c) growth phase, where surface development occurs, leading to the (d) synthesis of GNPs.

### 4.2 Silver nanoparticles (SNPs)

Before the development of nanotechnology, silver was thought to be just a metal. Later, it was found that it could be produced on a nanoscale. Modern engineering techniques for metallic silver resulted in ultrafine particles with distinct shapes and properties in the nanometre (nm) range. In the medical field, SNPs are used for their antifungal, antibacterial, anti‐inflammatory, antiviral, and osteoinductive properties. They can be synthesized by a variety of techniques, have optical properties, and accelerate the wound‐healing process. SNPs have great potential for cancer treatment. Several types of human cancer cells, including breast cancer cells, have been tested for the anti‐cancer effects of SNPs.^113^, ^114^ In recent times, some scientific studies have focused on the use of SNPs in combination with anticancer drugs to enhance antineoplastic efficacy, especially when used synergistically with the natural anticancer drugs used in their preparation.^114^ The chemical approaches are based on the chemical reduction of Ag+ ions by agents such as sodium borohydride, sodium citrate, sodium ascorbate, N,N‐dimethylformamide, and polymers, to name a few. The reducing agent produces metallic silver (Ag0) that aggregates into oligomeric aggregates. Colloidal metallic silver particles are formed in this manner. Chitosan, cellulose, and other polymers (polyethylene glycol (PEG), polyvinylpyrrolidone (PVP), and polymethacrylic acid (PMAA)) can be used as capping agents and surfactants as stabilizers to prevent the SNPs from aggregating to an undesirable size.^115^

Most plant extracts are used to synthesize SNPs offering therapeutic properties (anti‐inflammatory, anti‐cancer, etc.) that can be used in conjunction with the biological activity of SNPs. Some researchers have also suggested green‐synthesized SNPs as an antiangiogenic agent.^116^ The anti‐cancer activity of green‐synthesized SNPs is enhanced by the production of phytochemicals in response to the acidic environment of malignant cells.^117^ Excessive release of silver ions is observed at acidic pH. Selective death of cancer cells at acidic pH has been demonstrated by incubating SNPs in buffers at pH 5 and pH 7.4. Silver ions are also affected by how normal cells and cancer cells interact electrostatically. Extensive electrostatic interactions between normal and cancer cells are also required for the selective destruction of cancer cells. Another mechanism that has been described for the anticancer potential of SNPs is autophagy‐induced cell degradation leading to cell death. Autophagy is stimulated by green‐synthesized SNPs because the number of autophagolysosomes is increased, leading to the self‐destruction of cancer cells.^117^, ^118^ There are numerous methods for the conjugation of anticancer drugs with SNPs, including encapsulation, entrapment, and attachment to the surface of nanoparticles. For example, the incorporation of SNPs into the structure of liposomes improves stability, biocompatibility, and toxicity.^119^

Rozalen et al.^120^ prepared SNPs which were subsequently combined with methotrexate (SNPs‐ MTX). The antitumor activity of the free drug MTX, SNPs, and SNPs‐MTX was evaluated against a colorectal carcinoma cell line (HTC‐116) and a lung carcinoma cell line (A‐549). The anti‐cancer effect of SNPs‐MTX was more pronounced in the colorectal cancer cell line than in the lung cancer cell line, probably due to the absence of folate receptors in the lung cancer cell line.

By combining graphene oxide (GO) with methotrexate (MTX), Thapa et al. created a nanocomposite with SNPs. This nanocarrier combines the synergistic effect of SNPs, which increase the production of reactive oxygen species responsible for DNA damage and lead to apoptosis of graphene oxide, which can be used for photothermal ablation of tumours by absorbing near‐infrared radiation, and MTX, which has pharmacological activity against cancer.^121^

The anticancer drugs alendronate and doxorubicin were identified for coupling to SNPs^115^ and showed a significant synergistic effect against the HeLa cell line. Given the similarities between epirubicin and doxorubicin, it is reasonable to assume that the drug molecules formed coordinated covalent bonds with the SNPs. The synthesis of SNPs associated with doxorubicin. had a lower toxic effect on non‐cancer cells.^115^ SNPs modified with polyethylenimine (PEI) and functionalized with paclitaxel (PTX) reduced the growth of HepG2 cells and showed minimal cytotoxicity towards LO2 cells.^122^

SNPs were also used as nanocarriers for the anticancer drug gemcitabine. Cytotoxicity tests using silver and gold nanoparticles on breast cancer cell lines MDA‐MB‐453 showed that SNPs did not cause significant cytotoxicity in the breast cancer cell line.^123^ In the coming years, this area of research is expected to expand with a broader range of anticancer drugs and deeper information on the in vivo absorption, distribution, metabolism, excretion and toxicity of the silver nanosystems.^124^

#### 4.2.1 Synthesis of SNPs

**FIGURE 4:**
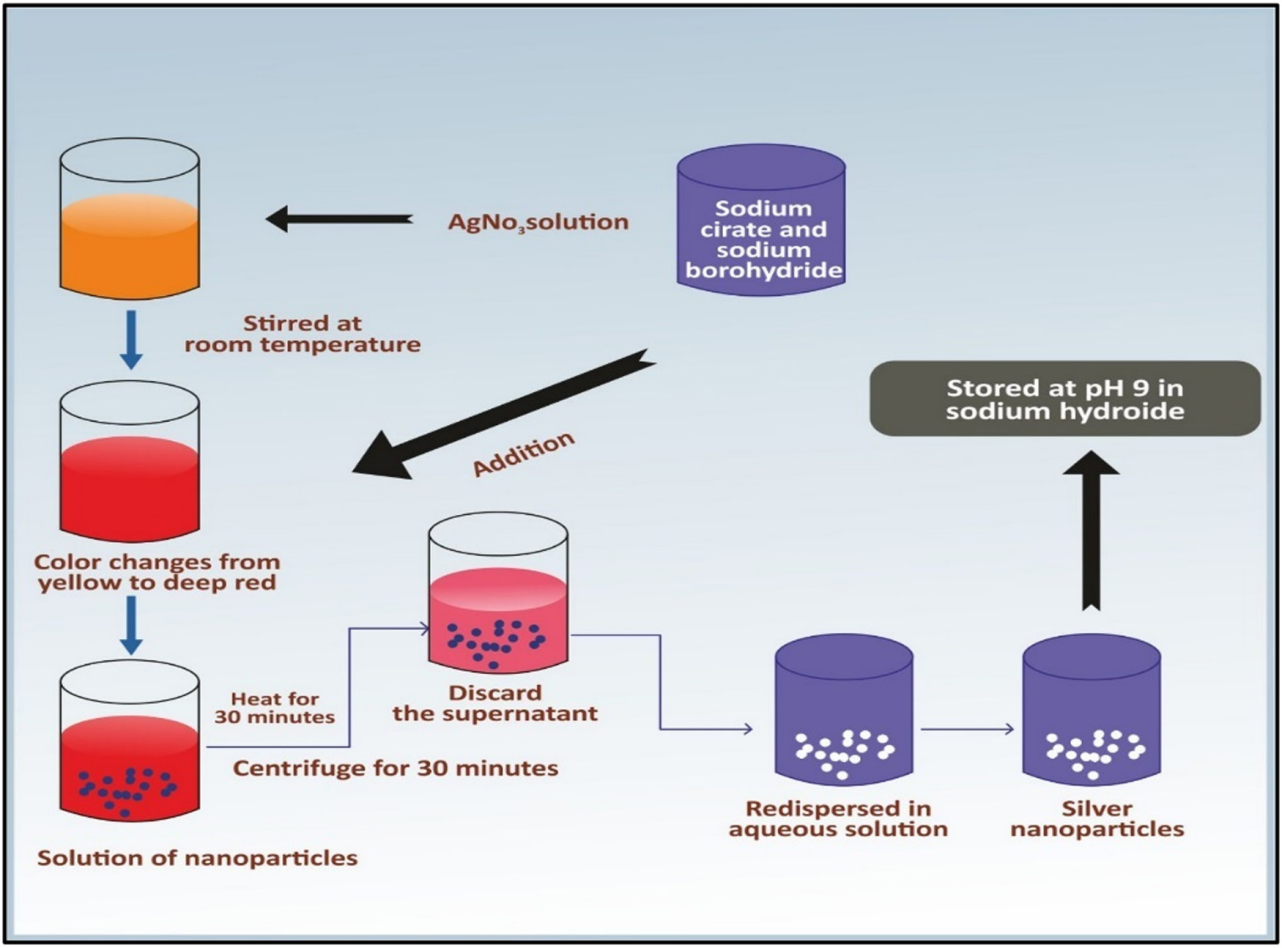
Step‐wise flowchart showing the synthesis of silver nanoparticles. The nanoparticle solution is prepared by adding sodium citrate and sodium borohydride, heated for 30 min, and then centrifuged for 30 min to separate the solution mixture into pellet and supernatant. The supernatant is discarded, whereas the pellet is dispersed in an aqueous solution to form the silver nanoparticles (SNPs).

Chemical, physical and biological methods have been used to produce silver nanoparticles.^125^ The common procedure for the preparation of silver nanoparticles is shown in Figure 4. The nanoparticle solution is prepared by adding sodium citrate and sodium borohydride solution, heated for 30 min and then centrifuged for 30 min to separate the solution mixture into pellet and supernatant, discarding the supernatant. The pellet is then dispersed in an aqueous solution to form the silver. The chemical and physical methods used to prepare SNPs are listed in Table 1.

**TABLE 1:** Representing the physical and chemical methods, which can be used for the synthesis of SNPs.

Chemical methods	Physical methods	Bio‐based methods
Tollens method^241^	Arc‐discharge method^242^	From bacteria, fungi, yeast, algae and plant^125^
Pyrolysis^243^	Evaporation‐condensation^244^	–
Photoinduced reduction^245^	Vapour condensation^244^	–
Electrochemical method^246^	–	–

## 5 NANOPARTICLES FOR TUMOUR TARGETING AND DRUG DELIVERY

Nanoparticles can be made from a wide variety of materials. These particles can be made in a variety of sizes and shapes, and their properties can change depending on the materials used in their manufacture. The materials, properties, and comments related to nanobiotechnology are listed in Table 2. In order to successfully deliver drugs to the desired site of a living organism to treat the disease without affecting other parts of the body, many different forms of drug delivery have been developed, such as polymer microspheres, lipid‐based carriers, liposomes, which include drug‐lipid complexes and lipid emulsions, and numerous other lipid‐targeted products.^126^, ^127^

**TABLE 2:** Review on nano‐biotechnology with their choice of materials, parameters and remarks.

S. No.	Type/Nature of material	Optimum parameters	Efficiency of the process	Remarks	References
1	Nanomaterials Nanoelectronics Nanomanufacturing and tools bio‐nanotechnology	Accuracy is equivalent to 1.0 but several other parameters go negative for nanomaterials, nanomanufacturing and nanoelectronics.	The research of the nanotechnology is evaluated with the help of statistical method to show the composition and accuracy of material.	This paper highlights the gaps and limitations in nanotechnology for the precise study and analysis of nanotechnology domains.	^247^
2	Materials for endodontic regeneration Teeth Aesthetic Materials Dendrimers and dendritic copolymers Dendrimers Nanocomposites	Whilst studying the nano‐dentistry, a nanostructured material used is sapphire, which is a type of composite material that increases durability and appearances. Sapphire is also used for the replacement of the upper enamel layer by artificial materials due to the material property which shows the property as 200–300 hardness than the ceramic.	The most suitable method for treatment as aesthetic dentistry procedure, dentition re‐naturalization is the most popular procedure in the dental practice.	The involvement of nanotechnology in the dentistry gives rise to new ideas for the oral health care remedies by preventing the cause rather than restoration or prevention.	248
3	Organic dendrimers Hollow polymer capsules Nano‐shells	Drug delivery is one of the potential applications against the application towards the treatment and remedy of several illnesses. Several diseases do not have cure nowadays but found the cure with help of nanotechnology and its future study will lead the potential benefit in medicine.	The nano‐formulations provide protection against several disease‐causing organisms by denaturation and degradation when exposed under extreme pH. It also increases half‐life of the drug due to its retention ability and only targeted release of drug at specific site.	The drawback of the nanoparticle at several cases is its toxicity towards gastrointestinal infection and bowel region diseases. The toxicity is identified and related to the several inflammatory mediators, which lead to the organ damage and inflammatory diseases.	248
4	Sonication French Pressure cell extraction Freeze thawed liposomes	Drugs that are lipophilic and amphiphilic have improved solubility. Passive targeted of immune system cells, particularly those of the mononuclear phagocytic system. Liposomes given systemically or locally with a sustained release mechanism. Mechanism for avoiding a specific location.	Several compounds, such as glycolipids or sialic acid, have been used to create liposomes with changed surfaces. Liposomes with improved drug delivery to disease sites due to their capacity to stay in circulation for lengthy periods of time are increasingly gaining clinical acceptability.	Liposomes with improved drug delivery to disease sites due to their capacity to stay in circulation for lengthy periods of time are increasingly gaining clinical acceptability.	249
5	Dendrimer Nanoshell Nanoparticle Polymer micelle	Cellular uptake has increased. Delivery that is sustainable, managed and targeted. P‐glycoprotein inhibition.	This focus upon nanoparticles ability to recognize cells using diverse ways that have unique identifying qualities that set them apart from past anticancer medicines.	Traditional chemotherapeutic drugs work by killing quickly dividing cells, and that is what neoplastic cells are best at doing so.	250
6	Types of material used wood Cotton Ramie Sisal Valonia Tunicates Bacteria	Aspect ratio obtained 20–100 10–30 ∼12 ∼60 50–200 ∼100 2–100	The mechanical, optical, and rheological properties of cellulose nanocrystals are described, as well as their chemical structure, sources, chemical and physical separation processes.	To attain higher productivity and quality, the new industrial extraction procedures for obtaining cellulose nanocrystals in large quantities must be optimized.	251
7	Gold nanoparticles in diagnostics Peptides	Various cellular targeting peptides have been conjugated to gold nanoparticles with a size of 20 nm to produce functional nanoparticles that penetrate the biological membrane and target the nucleus. Various nanoparticles have also been used to diagnose and treat cancer as drug delivery vehicles and targeted biomarkers.	Mixing alkane thiol‐terminated oligonucleotides with citrate‐capped GNP yields nanoconjugates densely functionalized with synthetic oligonucleotides. By forming a gold–thiol link, oligonucleotide ligands dislocate the citrate from the GNP.	A systematic programme is necessary to expose the relationships between experimental parameters (method, model, doses) and particle parameters (shape, size and with different molecular probes).	252
8	HA‐m‐poly(DEGMA‐co‐ CMA)	Under comparable conditions, a homopolymer poly (DEGMA) without photoactive CMA units was also synthesized. Because of the addition of hydrophobic coumarin moieties, the cloud point temperature of the poly (DEGMA‐co‐CMA) copolymers was lower than that of poly (DEGMA) (Tcmp −24°C).	Characterization and synthesis using a temperature‐triggered self‐assembly technique, as well as successful inclusion of weakly water‐soluble compounds and light sensitivity. A considerable shift in the threshold aggregation temperature after light irradiation, effective internalization by cancer cells overexpressing the CD44 receptor of HA, and the capacity to circulate for an extended length of time are all examples.	Temperature‐induced self‐assembly of dual stimuli (light and temperature)‐responsive HA derivatives produced by grafting CMA‐based copolymer chains on the polysaccharide and DEGMA backbone resulted in these gel particles.	253
9	Nanogels Micelles Nanofibers Liposomes	CS is a cationic linear polymer derived from the breakdown and N‐deacetylation of chitin. CS has been produced and used as a high‐value biomacromolecule, especially in biomedical applications.	To create unique CS derivatives with specific benefits, as well as various kinds of nanomaterials recently created from CS.	CS has excellent compatibility as well as biodegradability, immunogenicity and low toxicity indicating that it could be used as an ideal drug delivery mechanism.	254
10	DNA Microarray DNA Nanoflower DNA Dendrimers DNA Hydrogel	In comparison to other nanomaterials such as metallic, lipid‐based, organic, polymeric nanoparticles. The simplicity of DNA's base pairing concept enables for the creation of an infinite number of nanostructures of various charges, sizes, modularity and shapes to fulfil various functions.	Hydrogel, dendrimer and DNA nanoflower are three new but potential cancer theragnostic concepts. Individual formats' utility and promise in biomedical science, particularly in cancer therapy, will be examined.	The ability to easily assemble DNA structures into well‐defined designs susceptible of traversing physiological barriers and remaining stable in biological fluids inside the body, as well as the possibility for bio‐sensing and nanoscale precision tasks on target cells. If effective, it could provide a never‐before‐seen potential for early cancer identification and treatment at the level of a single cell.	255
11	Liposomes Polymeric nanoparticles Nanorods	The most commonly explored nanotechnologies in this field are polymeric nanoparticles, gold nano‐shells and liposomes with chemotherapy and PDT/PTT being the most widely used therapeutic methods.	The many types of nanotechnology employed in the treatment of oral cancer, as well as a summary of nanodrug administration routes.	Furthermore, because most researches are performed in animal models, overall safety of nanotechnology‐based medication delivery systems is uncertain.	256
12	Nanoparticles Nanocarriers Apoptosis‐induced gene therapy	Vector stability and transfection efficiency have been improved (SK‐BR3) and growth has decreased (MCF‐7).	The method of nanoparticle‐targeted drug delivery, as well as recent advancements in nanoparticle‐based carriers for microRNA, gene augmentation therapy and small interfering RNA in breast cancer.	However, despite ethical and safety problems, nanodevices remain a promising tool to fight over illness.	167, 257

### 5.1 Polymeric nanoparticles

Nanoparticles in their polymeric form must be absorbed by the body's circulatory system before they reach their target in the body, which may be malignant or healthy tissue. The mobility of nanoparticles is often influenced by their surface properties and dimensions. Often, the mononuclear phagocyte system (MPS) absorbs most of the injected nanomaterials, preventing them from reaching the disease target sites. In this case, this shortcoming is exploited by administering nano‐antioxidants that are preferentially absorbed by the liver to avert hepatic ischemia–reperfusion injury (IRI), a disease caused by reactive oxygen species (ROS).^128^ To prevent macrophages from ingesting the nanoparticles, a coating of polymers—including hydrophilic polymers—is applied to the surface of the particles. To prevent the nanoparticles from being degraded by enzymes, the biocompatibility of the particles can be increased by making them more water‐soluble and less susceptible to enzymes.^129^ Because of their biocompatibility, polymer‐based approaches have become increasingly popular in recent decades as a means of delivering anticancer drugs.^130^ These polymers are commonly used to physically dissolve, covalently bind, and entrap drugs needed to treat a specific disease or injury. Micelles and dendrimers are just two examples of the many different types of molecules that can be synthesized. Albumin, chitosan, heparin, poly‐[L‐glutamate], poly‐[D,L‐lactide‐co‐glycolide], polyethylene glycol (PEG) and many others are used to enhance drug delivery.^131^

### 5.2 Lipid‐based nanoparticles and liposomes

Due to their exceptional biological adaptability and biocompatibility, liposomes are widely used as drug carriers in the pharmaceutical industry. Liposomes are spherical vesicles composed of a lipid bilayer surrounding an aqueous core. These artificial membranes can be made from natural materials such as phospholipids and function similarly to biological cell membranes. Liposomes can encapsulate hydrophilic substances in their aqueous core or integrate lipophilic and amphiphilic drugs into their phospholipid membrane. Therefore, liposome formulations can improve the safety and efficacy of drug delivery at the site of action.^132^ Lipid‐based NPs, also known as LBNPs, are a particularly important type of nanoparticle for cancer therapy due to their breadth and diversity. Liposomes are widely used not only because they come in a variety of shapes and sizes, but also because they are highly biocompatible and can contain a wide range of cargoes. Some LBNPs (including Doxil® and Abraxane®) are already approved for the treatment of breast cancer, whilst others are currently being tested in clinical trials.^133^ Nanoparticles and lipid‐based liposomes have been used in the development of various types of anticancer drugs.^126^ In water, they form vesicles in which anticancer drugs can dissolve and remain stable once loaded into their structure. They can encapsulate both hydrophobic and hydrophilic substances. In addition to phospholipids, other substances such as cholesterol can be added to their formulations, which decreases the fluidity of the nanoparticles and increases the permeability of the hydrophobic drugs through the bilayer membrane, which improves the stability of these nanoparticles in the blood.^134^ Several researches have been conducted to make these types of anticancer drugs more suitable and tolerable for active targeted treatment. For example, liposomal formulations have been shown to improve the pharmacokinetics and pharmacodynamics of related drugs, such as doxorubicin.^135^ There are a few different methods by which liposomes can be successfully produced. These methods include the use of mechanical processes, the use of organic solvents, and the elimination of detergents from phospholipid/detergent‐micellar combinations. The final liposome structure is influenced by the preparation method, the amount and type of phospholipid used, the ionic and charge properties of the aqueous medium, and the duration of hydration of the lipids.^136^ There are two general approaches to nanocarrier vectorization.^137^ One is passive targeting, in which liposomes enter tumour cells exclusively through the cell membrane. The other is active targeting, in which liposomes are used together with antibodies that can find tumour cells altered in their structure. Third, stimulus‐sensitive liposomes can be used. Using an external trigger, parameters such as temperature, pH or magnetic fields can be changed to control the release of an anticancer drug.^138^

To increase the stability of the anticancer drug docetaxel, liposomes modified with PEG and anacardic acid were used for encapsulation. a pH‐sensitive liposome coated with chitosan composed of a glycol derivative. These liposomes had terminal amine groups that resulted in a negative surface charge. This allowed the liposomes to interact with the positive charge of the acidic extracellular tumour medium. When DOX was introduced into these liposomes, a significant increase in the anti‐cancer effect of the treatment was observed.^139^ To increase the water solubility of the anticancer drug sorafenib, a gadolinium‐based macrocyclic contrast agent for magnetic resonance imaging (MRI) was covalently bound to a lipid. In vivo and in vitro studies on this method of anticancer drug delivery showed that this liposome can also be used as a contrast agent.^140^

A liposome modified with penetrating peptides and transferrin was developed for the administration of 5‐fluorouracil (5‐FU) for the treatment of brain tumours. In vivo experiments showed that this bifunctional liposome was able to deliver 5‐fluorouracil (5‐FU) to tumour cells, demonstrating its efficacy in the treatment of cancer.^141^ A modified liposome containing PEG and arginine‐rich cell‐penetrating peptides was developed by Deshpande et al.^142^ This liposome exhibited a targeted ligand for interaction with the tumour. Encapsulation of Doxorubicin (DOX) in a liposome has been shown to increase release and efficacy in vivo whilst reducing citoxicity in vitro.^143^ DOX has also been encapsulated with curcumin (CUR) in a liposome designed for a long circulation time.^144^ Tian et al prepared liposomes by encapsulating PTX in a modified liposome containing hyaluronic acid, a biodegradable and biocompatible molecule that interacts with a specific ligand in tumour cells.^145^

### 5.3 Solid lipid nanoparticles

Solid lipid nanoparticles are a novel colloidal drug delivery technology composed of endogenous lipids that are solid at both room temperature and core body temperature. The size of these particles ranges from 50 to 1000 nm. Solid lipids such as mono‐, di‐ or triglycerides, fatty acids and complex glyceride mixtures are used as a matrix material for drug encapsulation. This matrix is held together by a combination of surfactants and polymers. SLNs offer a number of advantages, such as site‐specific targeting, long‐term physical stability, ability to regulate the release of both lipophilic and hydrophilic drugs, protection of labile drugs, low cost, ease of manufacture and non‐toxicity.^146^ In addition, SLNs have remarkably low toxic effects on human granulocytes. The combination of these significant advantages makes them a promising option for drug delivery systems. In contrast, SLNs have several disadvantages, such as lower drug uptake capacity and release of drug crystals under storage conditions.^146^, ^147^ The incorporation of active anticancer drugs into SLN formulations has been extensively explored because SLNs have the potential to increase oral drug bioavailability, preserve labile anticancer drugs, and reduce the dose without compromising efficacy.^147^, ^148^ For example, niclosamide‐loaded SLNs were developed to increased cell uptake and anticancer activity against triple‐negative breast cancer (TNBC) cells.^149^ Talazoparib loaded on SLNs was also developed, which increased the therapeutic index of the drug against TNBC cells. This improvement resulted from the reduction of toxicity and the overcoming of homologous recombination‐mediated resistance (HR).^150^, ^151^

SLNs containing resveratrol has been used to cure human breast cancer cells. Compared with free resveratrol, the authors of this study discovered that resveratrol SLNs had a significantly higher capacity to suppress cell growth. In addition, resveratrol SLNs showed a much more robust inhibitory effect on cell invasion and migration, suggesting a promising therapeutic potential in breast cancer (BreC).^152^ Another study showed that SLNs can be used as carriers to promote the efficacy of floxuridine in cancer therapy by allowing better cellular uptake of the drug. In this situation, the inefficient cellular absorption of the free drug could be remedied by loading SLNs with the lipophilic prodrug floxuridine.^153^ SLNs loaded with the hydrophobic anticancer drug indirubin from traditional Chinese medicine were used to treat human glioblastoma cells. The anti‐cancer effect of the hydrophobic drug was greatly improved when these SLN‐based formulations were tested in vivo.^154^

### 5.4 Nanostructured lipid carriers (NLC)

The second generation of lipid‐based nanocarriers, known as NLCs, has evolved from SLNs composed of both solid and liquid lipids. This technology was developed to overcome the limitations of SLNs. NLCs have a greater capacity for drug absorption and, because of the liquid lipids in the NLC formulation, can prevent the drug from being excreted during storage.^138^, ^155^ Unlike SLNs, which consist solely of solid lipids, NLCs contain both solid and liquid lipids such as glyceryl tricaprylate, ethyl oleate, isopropyl myristate and glyceryl dioleate. The average particle sizes, which vary depending on the type of lipids included and the manufacturing process, are very close to those of SLNs and typically range from 10 to 1000 nm. The main advantages of these nanoparticles are their ability to be loaded with hydrophilic and hydrophobic drugs, their ability to be modified at the surface, their suitability for site‐specific targeting, their ability to regulate drug release, and their low toxicity in vivo.^138^ However, there are still certain drawbacks, such as the release of the drug during the polymorphic transformation of the lipid from the nanocarrier matrix during storage and the low loading capacity.^156^

Fluvastatin, when coupled with lipoic acid and ellagic acid in a non‐liposomal formulation (NLC), could be used as a candidate for the treatment of prostate cancer because the combination promotes cell death compared to free drugs.^157^ NLC was used to enhance the bioavailability of lipophilic drugs such as thymoquinone (TQ). In this study, the anticancer effect of TQ‐NLC, a colloidal drug carrier, was demonstrated in Hep3B liver cancer cells.^158^ The case of artesunate nanoparticles modified with hyaluronic acid and cell‐penetrating peptides was particularly interesting because it showed that these nanoparticles were able to effectively identify and penetrate the tumour cell membrane, leading to very effective results against HepG2 cancer cells.

NLC loaded with orcinol glycoside and coated with PEG, a nanoformulation with oral delivery capability, showed anticancer activity against gastrointestinal cancer cell lines and hepatomas.^159^ NLCs loaded with 6‐gingerol were developed to increase the water solubility and oral bioavailability of bioactive 6‐gingerol, which is poorly soluble in water.^160^

## 6 TARGETED DELIVERY OF THERAPEUTIC NANOPARTICLES

New discoveries in biomedical sciences have led to the development of more effective therapeutic drugs. However, a key challenge that must be overcome before the efficacy of treating various diseases can be maximized is the transport of therapeutic chemicals to the target site. Non‐selectivity, undesirable side effects, limited efficacy and poor biodistribution are just some of the problems encountered when using conventional drugs. Therefore, researchers are focusing on developing systems that are both highly regulated and can serve multiple functions. The use of nanoparticles with tailored physicochemical and biological properties to deliver different chemicals to specific sites in the body is an exciting prospect. When a drug is successfully directed to a desired site and accumulates mainly there, it is called ‘targeted delivery’. For efficient targeted delivery, the drug‐loaded system should remain in the physiological system for an optimal period of time, escape the immunological system, target a specific cell/tissue and release the loaded therapeutic agent.^161^ There are two types of targeted delivery of therapeutic nanoparticles, namely passive and active targeting.

### 6.1 Passive targeting

The term ‘passive targeting’ refers to a method that takes advantage of enhanced retention (EPR), tumour microenvironment, and permeability effects to exploit the inherent size of nanoparticles and the specific vascular properties of the tumour. This approach has the potential to significantly improve both the bioavailability and efficacy of the drug. The EPR effect on angiogenesis is an essential component of tumour development. The 600–800 nm wide interstitial spaces between adjacent endothelial cells are enabled by the presence of angiogenic blood vessels in tumour tissue. Nanoparticle accumulation in tumours is determined by a number of factors, including nanoparticle surface properties, nanoparticle size, nanoparticle half‐life in the bloodstream and the extent of angiogenesis in the tumour. In two different models of polymeric nanoparticles, one decorated with the GE11 peptide for active targeting to the epidermal growth factor receptor (EGFR) and the other not, EGFR‐mediated intracellular internalization resulted in a greater therapeutic effect compared to the formulation that was not targeted. Nanoparticle accumulation is usually quite low in malignant tumours that are either proangiogenic or necrotic. However, the mechanisms leading to increased nanoparticle concentrations in some areas of tumour tissue whilst leaving other areas unaffected are not fully understood.^162^, ^163^, ^164^

### 6.2 Active targeting

The use of binary structural conjugates in drug delivery systems makes it difficult to achieve a high degree of targeted specificity. Because selective transport of nanoparticles specifically targeting tumour tissue depends on the cancer microenvironment, tumour angiogenesis and EPR tumour effects, the primary therapeutic investigation of polymeric nanoparticles does not include targeted delivery. One way extravasated drugs accumulate in the interstitium of the tumour is inadequate lymphatic drainage. Another possibility is increased osmotic pressure in the interstitium, which causes drugs to leak out of cells and redistribute in some areas of the malignant tissue.^162^, ^163^, ^164^

## 7 NANO‐BIOSTRUCTURES

Nanobiotechnology is a discipline that bridges the gap between the technical and scientific worlds. Current research focuses on improving current methods for identifying and studying sensors, specific systems and nanodevices; developing new methods for producing nanomaterials from biological components such as proteins and oligonucleotides. Other areas of research include the modification of artificial nanostructures to regulate and monitor biological processes and the development of new methods to identify and study sensors, systems and nanodevices.^165^ For example, classic liposomes with magnetic nanoparticles (MLPs) were used as thermal mediators in magnetic/superparamagnetic hyperthermia (MHT/SPMHT) for noninvasive cancer therapy.^166^

### 7.1 Peptide structure

Peptides have the ability to discretely assemble into suitable nanomaterials and actively respond to the tumour microenvironment. This ability can be achieved by modifying the conditions of synthesis and non‐covalent interactions of peptides,^167^ which can be designed to have a high degree of reactivity to the environment in the tumor.^168^ Self‐assembling peptide nanostructures, for example, can dissolve in a mildly acidic environment by protonation and disulfide bond breaking and by interaction with glutathione (GSH). Self‐assembling peptides have the potential to be used as smart nanoplatforms in cancer treatment because they are very sensitive to pH and GSH.^169^, ^170^

On the basis of peptide binding, examples include amphiphilic peptides, peptide structures, peptide conjugates, construction of cyclic peptide structures using the alternative L and D forms of amino acids and various auto‐complementary ionic particles.^171^ Other cases are reported according to their mutual complementarity. For example between chemotherapy and immunotherapy or phototherapy.^172^, ^173^

### 7.2 DNA nanostructure

DNA has also proven to be a useful building material for the bottom‐up strategy of artificially building supramolecular nanostructures. Professionals in the field of nanotechnology are interested in structured DNA nanotechnology because of its self‐assembling properties, which are not only predictable but also controllable.^174^, ^175^, ^176^ The potential of DNA to be formed into a series of motifs or tiles keeps it in the sights of researchers interested in creating scaffolds or geometric structures in one, two or three dimensions. DNA triple helices were structurally dismantled and combined with short sections of G‐quadruplexes to form a 1‐dimensional composite that was then stretched. These DNA tiles have several potential applications in DNA nanotechnology, such as communication modules in larger nanostructures, sensors, logic gates, and more. One of the key innovations of this study is the introduction of the concept of a new class of purely guanine‐based ‘sticky ends’ that can be used to connect DNA units without Watson‐Crick base pairing.^177^ Due to its self‐assembling nature, DNA is undoubtedly an excellent molecule for the formation of various multidimensional nanostructures and the placement of useful molecules and materials.^178^

### 7.3 Nano‐biomedicine

Nanobiotechnology is poised to revolutionize biomedical potential worldwide in areas ranging from drug delivery to immune sensor applications. Virtually every area of medicine can benefit. Examples include cancer (nano‐oncology), neurological diseases (nano‐neurology), cardiovascular diseases (nano‐cardiology), bone and joint diseases (nano‐orthopaedics), eye diseases (nano‐ophthalmology), and infectious diseases.^179^ In terms of treatment, diagnosis, and monitoring, nanomedicine can regulate the biological system. This control includes the refinement of therapeutic agents and tools for pharmacological and therapeutic targeting. The advances that nanobiotechnology will bring in the areas of diagnosis, prognosis, treatment and prevention are just the beginning of the development of a wide range of applications for this technology.^180^

### 7.4 Nano‐biomaterials

The dimensions of nanoscale materials have been reduced as they have evolved. This is currently one of the most critical areas of research to be decided upon to evaluate the performance of these materials and their specific properties. Key properties include optical properties, capillary forces, conductivity, melting temperature, electron affinity, ionization potential, reactivity and surface energy. Nanoscience provides important opportunities for theory and computation to play a leading role in the discovery process, as experimental tools often provide an incomplete picture of the structure and/or function of nanomaterials, and theory can often fill in missing features that are critical to understanding what is being measured.^181^ Nanoparticles containing biomolecules are amongst the most commonly modified nanomaterials. New and increasing uses of bio‐nanomaterials include diagnostics, clinical applications, immunological labelling, orthopaedic applications and drug delivery systems.^182^ Laser processing is on the rise because the capability of laser nanojoining combines a high‐precision feature with a flexible production platform. This approach usually improves the electrical conductivity and mechanical properties of the nanoscale materials used in the assembly.^183^ Natural and man‐made accidents, as well as the natural ageing process, have driven the development of bio‐nanomaterials for rapid recovery and support in the form of orthopaedic implants. In recent years, there has been a significant increase in the number of research papers dealing with the use of nanomaterials with organic structures for the regeneration of bone, cartilage, skin or dental tissue.^184^ The use of organic nanostructures, whether natural or artificial, has several advantages in a number of dental disciplines such as implantology, endodontics, periodontology, regenerative dentistry, and wound healing. The nanostructures offer a number of advantages, including increased colloidal stability, improved dispersibility, and enhanced surface reactivity.^185^

### 7.5 Drug delivery

**FIGURE 5:**
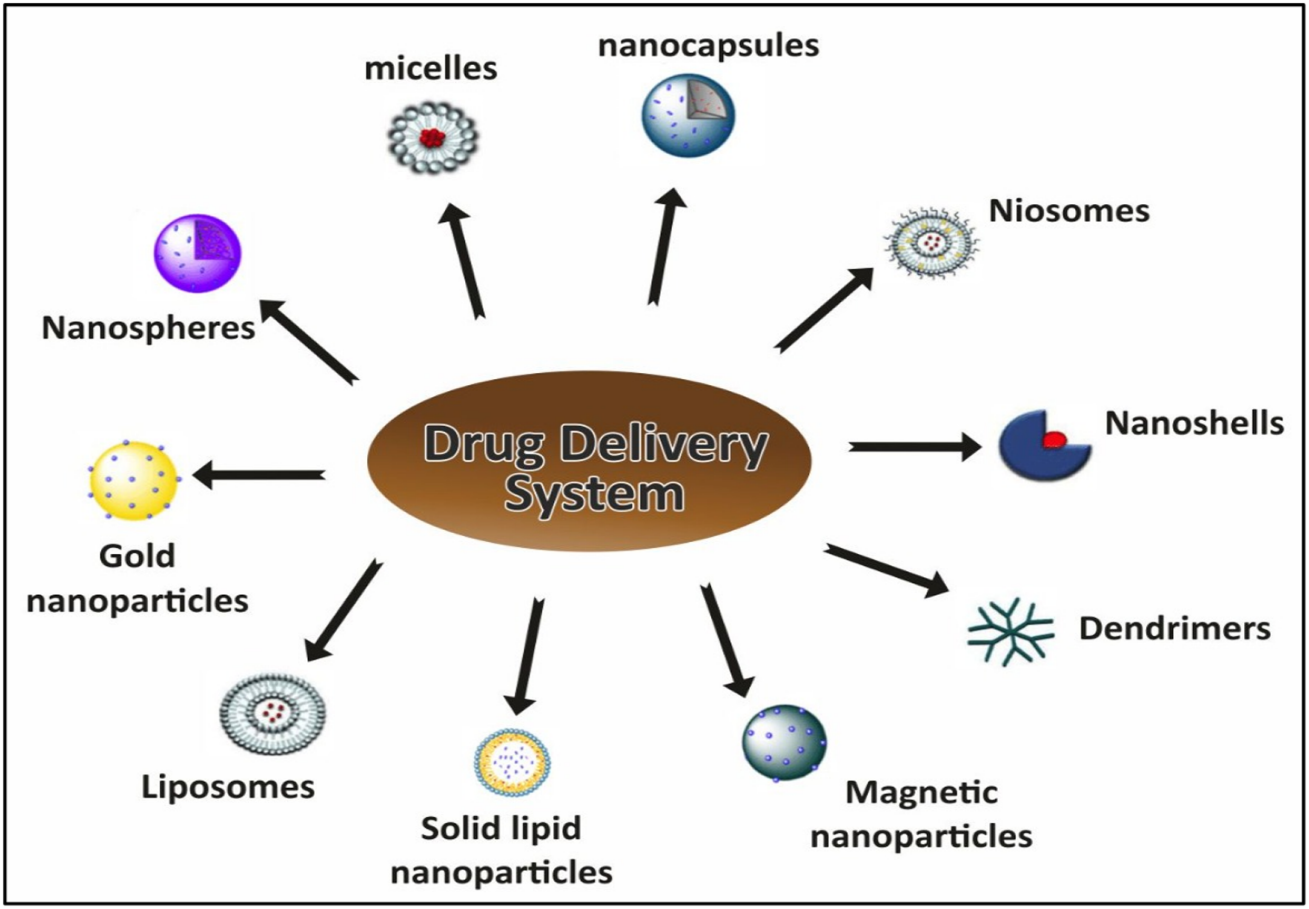
Diagram showing different drug delivery systems. There are number of drug delivery platforms including nanoshells, niosomes, dendrimers, magnetic nanoparticles, solid lipid nanoparticles, liposomes, gold nanoparticles, nanospheres, micelles, nanocapsules, nanocrystal, polymer‐drug conjugate, liposome, protein‐based nanoparticle and carbon nanotube.

Currently, there is a great interest in the application of nanobiotechnology as a means to develop new drug delivery systems and tools.^186^, ^187^ A diagram of the drug delivery system is shown in Figure 5. For example, many nanoparticles represent a promising class of drug‐delivery devices because they can be used in a controlled and targeted manner. The solubility and bioavailability of poorly soluble drugs can be improved by using nanosuspensions. Many problems in the formulation and administration of poorly water‐ and lipid‐soluble drugs can be solved by nanosuspensions. Paclitaxel/chitosan (PTX/CS) nanosuspensions have been proposed as a potential strategy for nanodrug delivery for cancer treatment.^188^ Research in nanobiotechnology also focuses on the application of magnetic properties in devices and the manufacture of materials.^189^ This field of research has a wide range of applications in medicine, the best known of which is magnetic separation and magnetic resonance imaging.^189^ Impedance biosensors are most commonly used to monitor markers of bone remodelling in osteoporosis, cytokinesis in cancer and to better understand neurological degenerative diseases.^190^, ^191^, ^192^, ^193^

### 7.6 Therapeutic diagnosis and treatment

Clinical diagnosis and treatment of a wide range of diseases could benefit from the application of materials that have been modified through the use of nanobiotechnology. Currently, a number of research initiatives are underway to develop a variety of innovative applications of nanoparticles that have amazing potential for drug uptake, release and therapy.^194^ Doxorubicin hydrochloride (DOX) was used as a model drug to evaluate the potential of Fe_3_O_4_ aqueous colloidal magnetic nanoparticles as a carrier system.^195^ To improve DOX accumulation, stimuli‐responsive (redox‐, light‐, enzyme‐ and pH‐sensitive) graphene oxide (GO) nanostructures were developed for DOX delivery.^196^ When comparing normal and malignant tissue, it should be noted that the activity of nanopolymer capsules helps to limit the release of drugs in an acidic environment.^197^ By increasing therapeutic efficacy and response and focusing treatment on more specific and sensitive body regions of the body, nanoparticles can be used to prevent tumour tissue destruction. Transition metal dichalcogenides or MoS_2_ have been used to fabricate a wide range of materials for electronics and medicine as an alternative to graphene. Due to its low cost and diverse properties, MoS_2_ is an excellent material for a variety of medical applications. MoS_2_ has very low natural solubility in water and is insoluble in acids, alkalis and some organic solvents. MoS_2_ nanosheets have remarkable absorption and photoconversion capabilities in the NIR region. Since biological tissue has no absorption in the NIR region, it is possible to use it for the development of cancer treatments (photothermal therapy).^198^

### 7.7 Nano‐biorobotics

Biomolecular research and advanced manufacturing techniques are contributing to the miniaturization of micro‐ and nanoelectronic devices. To target tumours and successfully alter the immunological microenvironment to suppress cancer, cell/bacterial components such as cell membranes, bacterial vesicles and other agents have been produced. The structural flexibility and natural properties of these native biomembranes make them sought‐after biological building blocks in nature and enable their integration into conventional synthetic materials. Examples include magnetotactic bacterial‐based biohybrid microrobots for combined tumour therapy by sequential magnetic/optical triggered delivery. Using magnetically driven *Magnetospirillum magneticum* and light‐guided indocyanine green nanoparticles, this bio‐microrobot can precisely target and ablate malignant tumours. The results of the study show that the bio‐microrobots can sequentially penetrate hypoxic tumours, where they can efficiently eliminate solid tumours using photothermal treatment guided by precise magnetic/light control.^199^ Bioactive materials based on cells and bacteria have enabled significant advances in cancer therapy, but significant obstacles remain before they can be used in a clinical setting. The heterogeneity of cancer results in a tumour microenvironment that is highly immunosuppressive and resistant to drugs, which can lead to deleterious off‐target effects and reduce the efficiency of bioactive materials in tumour control.^199^ Combinatorial or precise control techniques, such as multifunctional bioactive materials and programmable cell/bacterial microrobots, may be necessary for a successful cancer treatment plan to ensure the accuracy and efficacy of cancer treatments. The processes, toxicity, and immunogenicity of cell/bacteria‐based bioactive materials differ depending on their composition, size, surface chemistry, and shape. The manufacturing process of bioactive materials must be stable and reproducible, which requires reliable quality standards and control systems.^199^

### 7.8 Nano‐biodevices and electronics

The new technology and scientific field known as nano‐bio devices were created by the convergence of biological and synthetic materials and organic chemistry and biological research. Thanks to the functionalization and bioactivity of nanotubes, nanoparticles, and nanorods, researchers have been able to develop one‐, two‐ and three‐dimensional architectures of composite nanostructures on a variety of surfaces. In recent studies, some researchers have developed the idea of a nano‐biosensor activation system demonstrating single‐electron transistors, nano‐photoswitches based on photoelectrochemistry as well as electrochemiluminescence. Bioelectronic devices and systems can be significantly improved by combining nanoparticles and biomaterials in hybrid architectures. This is particularly true for biosensing applications. Biomaterials such as enzymes, DNA molecules, proteins or antibodies/antigens could be combined with semiconductor nanoparticles to create optical‐electrical biosensors that are both durable and sensitive. For example, various chemical substances, ions, biomolecules, and even whole cells or tissues can be detected with biosensors specifically designed for use in cancer research. Because carbon dots enhance fluorescence or enable measurement of lower enzyme concentrations, carbon dot‐based biosensors can be used as a platform to improve the measurement of enzyme activities associated with cancer and other diseases.^200^, ^201^ Colorectal cancer detection has benefited greatly from nanomedicine's increased sensitivity, lower cost, and reduced over‐ and underdiagnosis. Since colon malignancy cannot be fully detected at the molecular level, nanomedicine has replaced endoscopic analysis for morphological examination of the intestinal epithelium. Because ‐L‐fucose is a sugar overexpressed on the surface of colorectal cancer polyps, conventional endoscopy has been complemented by enteral administration of a mesoporous silica nanoparticles‐based formulation that actively targets ‐L‐fucose and is loaded with fluorescein (FITC) to optically visualize early colorectal tumour lesions.^202^

## 8 SEVERAL APPLICATIONS OF NANO‐BIOTECHNOLOGY

**FIGURE 6:**
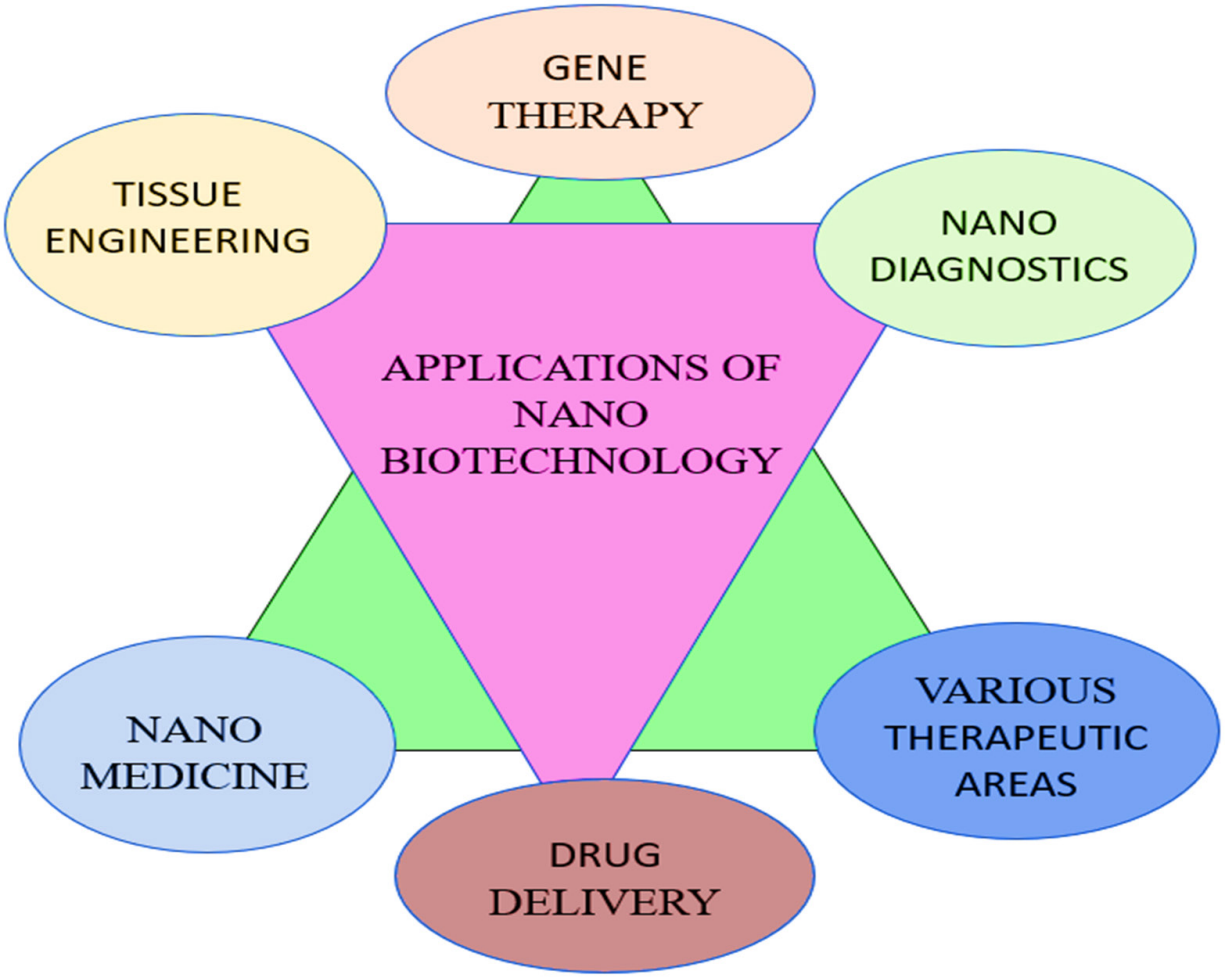
Diagram showing various applications of nano‐biotechnology. As an integrated branch of biology, there are different areas where it is vested involvement is warranted including nano‐medicine, nano‐diagnostics & detection, nano‐devices, tissue engineering, gene therapy, drug delivery, targeted therapy and molecular imaging.

Applications of nanobiotechnology include tissue engineering, drug delivery, nanomedicine, nanodiagnostics and a variety of therapeutic areas (Figure 6).^203^ In addition, there are a number of other applications listed below:

### 8.1 Cancer therapy

To maximize the efficacy of therapeutic molecules, it is necessary to distribute drugs at the right time and the desired target site. If the concentration is increased beyond the recommended level, it can be lethal. Because of the increased toxicity of cancer treatment, which can lead to severe side effects, these requirements are even more important. In recent years, numerous drug delivery techniques, including micro‐ and nanosystems and polymer conjugation, have been improved. Taken together, these technologies would improve the efficacy and safety of drug delivery, conventional chemotherapy, and the pharmaceutical and biomedical industries in cancer therapies. The development of design and synthesis of functional nanomaterials with high biocompatibility, selective targeting and excellent biodegradability is critical for their application in clinical cancer therapy. Modification of polymers is one way to increase the biocompatibility of nanomaterials. Second, the surface of the material can be modified to improve its biological targeting properties by adding chemicals that interact with tumour cells. Finally, the biodegradability of nanomaterials used in biomimetic fabrication and modification of nanomaterials for controlled cancer detection and therapy can be improved by using biomolecules such as proteins, DNA and peptides.^204^, ^205^, ^206^ In recent years, there has been a lot of buzz about a new type of cancer treatment called gas therapy (GT). Nitric oxide (NO), carbon monoxide (CO), hydrogen (H2), hydrogen sulfide (H2S) and sulfur dioxide (SO2) are just a few examples of gas molecules used to treat cancer by either directly killing tumour cells, increasing drug accumulation in tumours or making tumour cells more sensitive to chemotherapy, photodynamic therapy, or radiation therapy. Although gas treatment has come a long way, most gas molecules still have significant toxicity to normal tissues when administered systemically due to their nonspecific distribution.^207^, ^208^, ^209^ In order for gas treatment to be used in therapeutic areas, the biggest challenge is figuring out how to target the gas molecules for release in the affected areas. Near‐infrared (NIR) light is widely used to initiate the cleavage and release of gasses from nanoscale agents through photothermal or photodynamic effects, resulting in the on‐demand release of gas molecules with high controllability. NIR light is a special non‐invasive stimulus with deep penetration.^210^

### 8.2 Development of nano‐polymers

Smart nano‐polymers are the most commonly used polymers for targeted and precise drug delivery. The properties of linear smart polymers and matrices are determined based on side chains and reactive functional groups. Occasionally, it is necessary to remove components that adversely affect tissue and bone. Nanobiotechnology plays a key role in the development of various nanoparticle polymers, such as fullerenes and dendrimers, which are used for gene and drug delivery. Apart from tumour suppression, functionalized fullerenes have properties that counteract systemic toxicity and drug resistance typical of conventional chemotherapeutic approaches.^211^, ^212^ Certain fullerenes have been shown to enhance the chemosensitization of tumour cells to chemotherapeutic agents in drug‐resistant cancer cells.^213^, ^214^ Dendrimers are nanoscale molecules that are radially symmetric and have a well‐defined, homogeneous, and monodisperse structure. They usually consist of three parts: a central core, an inner dendritic structure (the branches) and an outer surface with surface functional groups.^215^ The combination of these elements results in products of different shapes and sizes with protected inner cores that have promising applications in the fields of biology and materials science. The properties of dendrimers are often determined by the functional group associated with the molecular surface, as it affects solubility and chelation. However, the nuclei also impart special properties to the cavity size, absorption capacity, and trapping and release properties. Due to their properties such as hyperbranching, well‐defined globular structures, excellent structural homogeneity, multivalence, changeable chemical composition and great biological compatibility, dendrimers are attractive candidates for use in biomedical applications.^215^ In the field of biomedical research, dendrimers are rapidly becoming a remarkable technique for drug delivery. In recent years, many dendrimer‐based formulations have been developed for the treatment of breast cancer (BC). Dendrimers have shown promise in mitigating some of the drawbacks of the standard treatments currently used for BC. The next generation of BC drugs may consist of dendrimers containing active agents, targeted ligands, and molecular imaging.^216^ Dendrimers may also be useful for other purposes, such as gene transfection to deliver drugs in the form of combinations to specific tumour sites with increased efficacy and less damage to off‐target organs.^217^ Other polymers, such as those made from lactic acid, have long been used to encapsulate drugs such as tamoxifen to reduce tumour size in a rat model of breast cancer.^218^

### 8.3 Antimicrobial bandages

Most conventional wound dressings are ineffective and suffer from limitations such as inadequate antibacterial activity, toxicity, insufficient moisture delivery to the site and poor mechanical performance. The use of poor wound dressings can delay the wound‐healing process. To address this need, scientists have developed antibacterial wound dressings using silver nanoparticles (SNPs).^219^, ^220^ The silver ions prevent microorganisms from absorbing oxygen. In other words, when silver comes into contact with dangerous cells, it suffocates them and destroys them. Bacteria in a biofilm can secrete extracellular polymers that make them resistant to antibiotics. Bacterial biofilm is the greatest obstacle to recovery. It can develop either spontaneously or as a result of other factors. After an intensive surgical procedure, such as treatment for endometrial cancer, a patient may become colonized with various types of bacteria, and may need to stay in the hospital longer.^221^ It is possible that the usage of antimicrobial bandages might improve several wound healing factors.^222^, ^223^

### 8.4 Nano‐filters

The separation of molecules such as proteins and DNA is a possibility for nanoscale genomics‐based filtering research. It also protects against numerous biological perturbations, such as viruses up to 30 nm in diameter.^224^

### 8.5 Nanobeads

These are polymer beads with a diameter of 0.1 to 10 micrometres. The approach is to impregnate fluorescent crystal chips in nanobeads to make the move and establish the next level of disease therapy and diagnostics by enabling 1000 of biological interactions to jointly improve clinical diagnosis and drug development. Nanocrystals, nanodots and even quantum beads are sometimes used interchangeably.^225^

### 8.6 Nanobiotechnology in the food sector

Nanotechnology applications for food and agriculture use nanosensors for the detection and quantification of organics, pathogens and food composition and other chemicals; high‐performance sensors (electronic tongue and nose) and thin films for the preservation of edible fruits^226^, ^227^

### 8.7 Gene delivery

Recent challenges in gene therapy include effective manufacturing and pharmaceutical processing, and the possibility that a generated mutant could reverse function. Another problem with the use of viral vectors for gene delivery is their potential immunogenicity. In this context, nanotechnological methods for gene therapy in humans have been explored and nonviral vector nanoparticles, typically 50 to 500 nanometres in size, have been developed as carriers for plasmid DNA.^228^ Nanoparticles with non‐viral vectors can help replace defective genes in humans by successfully introducing less immunogenic gene carriers instead of problematic viral vectors.^228^, ^229^, ^230^

### 8.8 Liposomes

Various forms of liposomes can be used to deliver drugs locally and effectively to desired targets. In addition, liposomes are sometimes part of the targeted treatment of the diagnosed disease.^231^ For example, intravenous injection of Trojan liposomes (THL) containing a monoclonal antibody (Mab) against the human insulin receptor allowed the transport of a luciferase reporter gene plasmid into the brain of rhesus monkeys. To produce THLs, plasmid DNA was encapsulated in pegylated liposomes and coupled with a Mab targeting an endogenous receptor expressed on both the blood–brain barrier (BBB) and brain cells. The specific Mab could act like a molecular Trojan horse by binding to the endothelial receptor and initiating its transport across the BBB. Some of these successful studies could be the reference point for the future towards targeted therapy and may add towards the need for more advancement in molecular medicine. This technology would allow nanometre‐sized constructs to be used to deliver the drug to the desired location with a longer half‐life to treat the disease.^231^, ^232^, ^233^

### 8.9 Biopharmaceuticals

Nanobiotechnology has the potential to develop therapies for diseases that cannot be treated with conventional drugs. Throughout its history, the pharmaceutical industry has focused on the discovery and development of new drugs to treat more than 500 different diseases. However, between 70% and 80% of drug development attempts fail. Millions of dollars are often lost because the investments in research and development were made too late. Nanobiotechnology allows us to regulate the position and timing of chemical processes and the physical manipulation of atoms, molecules and targets on solid substrates. This is done in a relatively short time and with minimal resources (reagents and solutions). This advance will reduce the cost of drug discovery, make a variety of chemical entities available and facilitate the development of highly specialized drugs.^234^, ^235^

## 9 RECENT ADVANCEMENT IN NANOMEDICINE AND DRUG DELIVERY

Nanomedicine is perhaps one of the most exciting areas of scientific research. Thousands of clinical trials have been conducted in recent years, and about 1500 patents related to nanomedicine have been filed during the same period. Several studies, some of which have not used medical devices, have shown nanomedicine to be an effective means of detecting and treating cancer. Non‐medical technologies have also been used in this research. In addition, the use of many different types of nanoparticles allows targeted delivery of drugs to damaged cells, such as cancer and tumour cells, without affecting cell function. Significant progress has also been made in the diagnostic use of nanoparticles of metals. The use of various metals, including gold and silver, in diagnostics and therapeutics could eventually lead to the broader application of nanomedicines. Soft tumours appear to absorb gold nanoparticles effectively and have radiation‐sensitive tumour‐based therapy (e.g. near‐infrasound) for selective eradication.^236^ Nanomedicine is clearly the direction in which research and development will move in the coming years and decades.^237^ Studies on materials with improved consistency, drug loading, and release capabilities would involve a further investigation. Although much research has been done to understand the potential future of nanomedicine and nanomedicine delivery systems, the actual impact of this field on medical systems is still quite limited. This is true even in the area of cancer treatment and diagnosis. This area of research is only about 20 years old, and there is relatively little significant research on this topic and few distinguishing features. One of the most fruitful avenues for future study will focus on identifying key indicators of diseased tissue. These include biological markers that allow absolute targeting whilst maintaining the integrity of the normal cellular process. As our understanding of molecular disease continues to increase, we will be able to better utilize nanomedicine or reflect a comparable marker size of nanomaterials subcellularly to open the door to new diagnostic and therapeutic methods. Determining the molecular fingerprints of diseases will lead to further advances in the application of nanomedicine. Beyond what has been covered in this analysis regarding nanoprobes and nanodiagnostic tools, there is an urgent need for additional research before the field of nanomedicine can be applied on a larger scale.^238^

Numerous studies in the emerging field of nanomedicine are investigating its potential for use in the early stages of biomedicine. Biomaterials and pharmaceutical formulations are the main research topics here. Animal studies and transdisciplinary investigations, which take a lot of time and resources, could provide valuable data that can be used for future applications such as diagnostic and pharmacological trials. As there is currently a global trend towards more accurate diagnoses and therapies, the development of nanodrug delivery systems and nanomedicine seems to have a promising future. However, no significant progress has been made in developing a controlled release of drugs at specific sites, technologies to assess these events, pharmacological effects at the tissue/cell level or theoretical mathematical prediction models. These are all issues that need to be improved in the future.^238^, ^239^

Whilst nanomedicine has many potential benefits, its potential hazards to humans and the environment must also be thoroughly investigated. Therefore, the acute and chronic toxic effects of novel nanomaterials on ecosystems and human populations need to be thoroughly investigated. Considerable scientific progress would be achieved if the price of nanomedicines decreased as their availability increased. The limitations described in the previous section will be addressed in the future as new applications of nanomedicine are developed.^238^, ^239^

## 10 CONCLUSION, CHALLENGES AND FUTURE PERSPECTIVES

Nanobioscience is an emerging field at the interface of biology, chemistry and physics that holds great promise due to the rapid development of medical technology. The rapid progress in medical technology makes the interdisciplinary field of nanobioscience an area with enormous potential in the fields of biological, chemical, and physical science. Medicine is making incredible advances that can be directly attributed to the use of nanobiotechnology. In this study, we examined nanobiotechnology as one of the most rapidly developing areas of scientific and engineering research, including applications for drug delivery and incorporation into the human body through the creation of bio‐scaffolds. Coating the receptor site of a nano‐biomaterial with a drug so that it acts only where needed to inhibit disease‐causing organisms in the human body, such as tumours and cancer, is an example of site‐selective drug delivery.^240^ The challenge with using metal‐based nanoparticles for drug delivery, such as gold and silver nanoparticles, is that they must be biocompatible, bioadaptable and site‐specific. Another challenge is the ability to penetrate the bloodstream barrier without affecting other body organs and to target infected body sites.

There is no single person, group or intellectual field capable of providing answers to the questions that nanotechnology will raise. Developing devices to assess human exposure to engineered nanomaterials in air and water is amongst the top five problems. Due to their potential importance in regenerative and diagnostic medical applications, there is a great need for research in this area. If diseased cells could be detected more quickly, perhaps even at the level of a single diseased cell, then urgent treatment could be given to the diseased cells, preventing the disease from spreading to other regions of the body and causing further damage.

## AUTHOR CONTRIBUTIONS

**Ambikesh Soni:** Investigation (equal); writing – original draft (equal). **Gagan Kant Tripathi:** Conceptualization (equal); supervision (equal); writing – review and editing (equal). **Priyavand Bundela:** Methodology (equal); supervision (equal). **Pradeep Kumar Khiriya:** Methodology (equal); resources (equal). **Purnima Swarup Khare:** Methodology (equal); supervision (equal); visualization (equal). **Abhijit Dey:** Investigation (equal); methodology (equal); supervision (equal); validation (equal). **Balachandar Vellingiri:** Formal analysis (equal); supervision (equal); validation (equal). **S. Suresh:** Supervision (equal); writing – review and editing (equal). **S. Arisutha:** Supervision (equal); validation (equal). **José Manuel M. Pérez de la Pérez de la Lastra:** Funding acquisition (equal); investigation (equal); supervision (equal); writing – review and editing (equal). **Manoj K. Kashyap:** Methodology (equal); writing – original draft (equal); writing – review and editing (equal). **Manohar Prasad Bhandari:** Methodology (equal); writing – original draft (equal); writing – review and editing (equal).

## FUNDING INFORMATION

This research was funded by ‘Agencia Canaria de Investigación, Innovación y Sociedad de la Información (ACIISI) del Gobierno de Canarias’ (Project ProID2020010134), and CajaCanarias (Project 2019SP43). MKK is the recipient of an extramural grant (sanction #: 5/13/55/2020/NCD‐III) from the Indian Council of Medical Research (ICMR), New Delhi, India. State Plan for Scientific, Technical Research and Innovation 2021–2023 from the Spanish Ministry of Science and Innovation (ProjectPLEC2022–009507).

## CONFLICT OF INTEREST STATEMENT

The authors confirm that there are no conflicts of interest. They have no known competing financial interests or personal relationships that could have influenced the work reported in this paper.

## Data Availability

All data generated or analysed during this study are included in this manuscript.
